# The *TERT* Promoter is Polycomb-Repressed in Neuroblastoma Cells with Long Telomeres

**DOI:** 10.1158/2767-9764.CRC-22-0287

**Published:** 2024-06-20

**Authors:** Mindy K. Graham, Beisi Xu, Christine Davis, Alan K. Meeker, Christopher M. Heaphy, Srinivasan Yegnasubramanian, Michael A. Dyer, Maged Zeineldin

**Affiliations:** 1 Department of Radiation Oncology and Molecular Radiation Sciences, Johns Hopkins University School of Medicine, Baltimore, Maryland.; 2 Department of Urology, Northwestern University, Feinberg School of Medicine, Chicago, Illinois.; 3 Center for Applied Bioinformatics, St. Jude Children's Research Hospital, Memphis, Tennessee.; 4 Department of Pathology, Johns Hopkins University School of Medicine, Baltimore, Maryland.; 5 Department of Oncology, Johns Hopkins University School of Medicine, Baltimore, Maryland.; 6 Department of Urology, Johns Hopkins University School of Medicine, Baltimore, Maryland.; 7 Department of Medicine, Boston University School of Medicine, Boston, Massachusetts.; 8 Department of Developmental Neurobiology, St. Jude Children's Research Hospital, Memphis, Tennessee.; 9 Howard Hughes Medical Institute, Chevy Chase, Maryland.; 10 Department of Ophthalmology, University of Tennessee Health Science Center, Memphis, Tennessee.

## Abstract

Acquiring a telomere maintenance mechanism is a hallmark of high-risk neuroblastoma and commonly occurs by expressing telomerase (*TERT*). Telomerase-negative neuroblastoma has long telomeres and utilizes the telomerase-independent alternative lengthening of telomeres (ALT) mechanism. Conversely, no discernable telomere maintenance mechanism is detected in a fraction of neuroblastoma with long telomeres. Here, we show, unlike most cancers, DNA of the *TERT* promoter is broadly hypomethylated in neuroblastoma. In telomerase-positive neuroblastoma cells, the hypomethylated DNA promoter is approximately 1.5 kb. The *TERT* locus shows active chromatin marks with low enrichment for the repressive mark, H3K27me3. *MYCN*, a commonly amplified oncogene in neuroblstoma, binds to the promoter and induces *TERT* expression. Strikingly, in neuroblastoma with long telomeres, the hypomethylated region spans the entire *TERT* locus, including multiple nearby genes with enrichment for the repressive H3K27me3 chromatin mark. Furthermore, subtelomeric regions showed enrichment of repressive chromatin marks in neuroblastomas with long telomeres relative to those with short telomeres. These repressive marks were even more evident at the genic loci, suggesting a telomere position effect (TPE). Inhibiting H3K27 methylation by three different EZH2 inhibitors induced the expression of *TERT* in cell lines with long telomeres and H3K27me3 marks in the promoter region. EZH2 inhibition facilitated MYCN binding to the *TERT* promoter in neuroblastoma cells with long telomeres. Taken together, these data suggest that epigenetic regulation of *TERT* expression differs in neuroblastoma depending on the telomere maintenance status, and H3K27 methylation is important in repressing *TERT* expression in neuroblastoma with long telomeres.

**Significance:**

The epigenetic landscape of the *TERT* locus is unique in neuroblastoma. The DNA at the *TERT* locus, unlike other cancer cells and similar to normal cells, are hypomethylated in telomerase-positive neuroblastoma cells. The *TERT* locus is repressed by polycomb repressive complex-2 complex in neuroblastoma cells that have long telomeres and do not express *TERT*. Long telomeres in neuroblastoma cells are also associated with repressive chromatin states at the chromosomal termini, suggesting TPE.

## Introduction

Telomeres are tandem hexanucleotide repeats located at eukaryotic chromosomal termini that in most normal somatic cells shorten with every replication cycle (1). Stem and progenitor cells express a reverse transcriptase called telomerase (TERT) that utilizes a long noncoding RNA template (*TERC*) to add telomeric repeats specifically to chromosomal ends to maintain telomeres (1) and prevent telomere-induced senescence and apoptosis (2). Expression of *TERT* is strongly repressed in most somatic cells, limiting the number of replications that a cell can undergo, referred to as the Hayflick limit, and protects against malignant transformation (3). To overcome this key replicative barrier, the majority of cancers express *TERT* to maintain telomere lengths (4). However, approximately 4%–11% of cancers rely on a telomerase-independent recombination-mediated pathway termed Alternative lengthening of telomeres (ALT) to maintain telomere lengths (5). In some rare cases, cancer cells with long telomeres are negative for both telomerase and ALT, suggesting either an undefined telomere maintenance mechanism, or a lack of a mechanism (6, 7).

Neuroblastoma is a pediatric tumor that arises from the developing sympathetic neurons and is one of the leading causes of cancer-related death in children (8). The acquisition of a telomere maintenance phenotype defines high-risk neuroblastoma and failure to maintain telomere lengths may lead to spontaneous regression of the tumor (9). Telomere maintenance in neuroblastoma is controlled by genetic and epigenetic factors through three mutually exclusive mechanisms (10, 11). First, amplification of the oncogene *MYCN,* which is the most frequent genetic alteration seen in high-risk neuroblastoma (12–14). MYCN is a transcription factor that induces transcriptional and epigenetic reprograming (15, 16) and *MYCN*-amplified neuroblastoma cells express *TERT* (17). Second, in a subset of high-risk neuroblastoma cases, rearrangement of the *TERT* locus on chromosome 5 results in a juxtaposition of a proximal enhancer and *TERT* that consequently induces a high level of *TERT* expression, a process termed “enhancer hijacking” (11). Finally, *ATRX* mutations, which are strongly associated with ALT, are frequently detected in high-risk neuroblastoma, especially in adolescents and young adults (16, 18, 19). ATRX is a chromatin remodeler, and its mutations are associated with widespread epigenetic alterations (16, 20–22). Taken together, these observations suggest that epigenetic factors play an important role in telomere maintenance and the modulation of *TERT* expression in neuroblastoma.

DNA methylation and posttranscriptional histone modifications are two major epigenetic mechanisms that control gene expression (23). Although extensively studied, the significance of the methylation of CpG islands in the *TERT* locus is unclear (24). Proximal promoter methylation is generally considered a repressive mark that limits the accessibility of genes to transcriptional factors (23). Paradoxically, *TERT* promoters are hypermethylated in telomerase-positive cancers and hypomethylated in most untransformed cells, suggesting that methylation is associated with telomerase expression in cancer (25, 26). However, many studies have consistently shown that indeed promoter DNA methylation inhibits *TERT* expression (27, 28).

In addition to DNA methylation, other epigenetic changes including posttranscriptional histone modifications control gene expression, with different combinations of chromatin marks defining distinct functional activities in the genome (29). Acetylation of histone H3 lysine at positions 9 and 27 (H3K9Ac, H3K27Ac) and trimethylation at amino acid 4 (H3K4me3) reduce chromatin packing and permit the binding of transcription factors, thereby marking actively expressed genes. Conversely, H3K9me3 and H3K27me3 are associated with closed heterochromatin and considered repressive epigenetic marks (23). These chromatin marks are installed and maintained by large multi-subunit protein complexes. For example, the polycomb repressive complex-2 (PRC2) plays an essential role in methylating H3K27 to ensure silencing of target genes (23, 30).

Recently, we characterized the epigenetic landscapes in a wide range of neuroblastoma cell lines, orthotopic patient-derived xenografts (O-PDX), and cancer tissues taken at the time of autopsy (16). In these samples, we performed whole-genome bisulfite sequencing (WGBS) and chromatin immunoprecipitation sequencing (ChIP-seq) for eight different chromatin marks and the epigenetic regulators: BRD4, RNA polymerase PolII, and the transcription regulator CTCF. These data were compiled into 18 different epigenetic states using Hidden Markov modeling (HMM). In addition, we mapped the genomic distribution of MYCN binding sites in the *MYCN*-amplified cells (16). Here, we used these data to comprehensively examine the epigenetic regulation of the *TERT* promoter region in neuroblastomas. We found that, similar to normal tissues of developing adrenal medulla, the *TERT* promoter is hypomethylated in all neuroblastoma cells we studied, independent of telomere maintenance status. However, in neuroblastoma cells with long telomeres, including ALT-positive cells, we observed that the hypomethylation of the *TERT* promoter extended throughout the *TERT* promoter CpG island. In contrast, the *TERT* gene body is hypermethylated in telomerase-positive neuroblastoma cells. In addition, telomerase-positive neuroblastoma cells have the active chromatin marks H3K9Ac and H3K27Ac in the *TERT* promoter, while the *TERT* promoter of neuroblastoma cells with long telomeres are silenced by H3K27me3, suggesting polycomb repression of the *TERT* locus. Indeed, pharmacologic inhibition of EZH2, the enzymatically active subunit of PRC2, resulted in an increase in *TERT* expression, suggesting derepression of the *TERT* locus. Altogether, these data contextualize the epigenetic regulation of the *TERT* locus in high-risk neuroblastoma based on differing telomere maintenance mechanisms.

## Material and Methods

### Cell Lines and Culture

U2OS (RRID:CVCL_0042), 293T (RRID:CVCL_0063), SKNBE2 (RRID:CVCL_0528), IMR32 (RRID:CVCL_0346), HTB11 (RRID:CVCL_0531), and SKNFI (RRID:CVCL_1702) cells were obtained from the ATCC. CHLA90 (RRID:CVCL_6610) and LAN6 (RRID:CVCL_1363) cells were obtained from the Children Oncology Group. NB-5 cells (RRID:CVCL_9890) were available at St. Jude Children's Research Hospital. SKNMM cells (RRID:CVCL_C8G1) were a generous gift from Dr. Nai-Kong Cheung from the Memorial Sloan Kettering Cancer Center while NBLS (RRID:CVCL_2136) cells were a kindly provided by Dr. Garrett M Brodeur from the Children Hospital of Philadelphia (CHOP). U-251 (RRID:CVCL_U251), MOG-G-UVW (RRID:CVCL_2614), and PC-3 cells (RRID:CVCL_0035) were purchased from Millipore. 293T, SKNFI, NB5, and SKNFI cells were grown in DMEM (Lonza, catalog no. 12-614F) supplemented with 10% FBS and 1X Penicillin/Streptomycin/l-Glutamine (Gibco, catalog no. 1037-016). NBLS, SKNMM, and LAN6 cells were maintained in RPMI1640 medium (Lonza, catalog no. 12-167Q) supplemented with 1X Penicillin/Streptomycin/l-Glutamine (Gibco, catalog no. 1037-016) and 10% FBS. IMR-32 cells were grown in Eagle Minimum Essential Medium cells supplemented by 1X Penicillin/Streptomycin (ATCC, catalog no. 30-2003) and 10% FBS while U2OS cells were grown in McCoy's 5A medium (Sigma, catalog no. M8403) supplemented with 1X Penicillin/Streptomycin (Gibco, catalog no. 15140-122) and 10% FBS. The cells were checked for *Mycoplasma* contamination using Universal Mycoplasma Detection Kit (ATCC, catalog no. 30-1012K) following the manufacturer's protocol and the identity of the cell lines were confirmed periodically using short tandem repeat profiling. Cells were used within the first five passages after thawing.

### FISH for Telomeres and MYCN

FISH was done as described previously (16). In brief, telomere interphase FISH was performed using fluorescein isothiocyanate–labeled PNA telomere probe (DAKO, catalog no. 5327) following the manufacturer's protocol. For *MYCN* FISH, *NMYC* BAC DNA (*RP11-1183P10*) and human chromosome 2 control (2q11.2) BAC DNA (*RP11-527J8*) were labeled with red-dUTP and green-dUTP, respectively, using nick translation kit (Molecular Probes, catalog no. AF594). The samples were denatured 90°C for 12 minutes. Hybridization with the labeled probes mixed with sheard human DNA was done overnight at 37°C on a ThermoBrite (Abbott, RRID: SCR_010477) in the hybridization solution [50% formamide, 10% dextran sulfate, and 2 × saline-sodium citrate (SSC) solution]. Excess probes were washed and 4,6-diamidino-2- phenylindole (DAPI; 1 µg/mL) was used as a counter stain. Image capture and analysis was done using Zeiss Axio Imager.Z2 microscope and GenASIs scanner (Zeiss).

### Telomere qPCR

Quantification of telomeric repeats was performed using qPCR as described previously (16). In brief, DNA was extracted using DNAeasy Kit (Qiagen, catalog no. 69504) following the manufacturer's instructions. qPCR reaction was done in 10-µL total reaction volume; containing 10 ng DNA template, 10 mmol/L primer mix, and 5 µL SYBR green Select master mix CFX (Thermo Fisher Scientific, catalog no. 4472942). Every reaction was done in triplicate and for every sample, two sets of primers were used; one for the telomeric sequence and the other for the control locus *RPLP0*. Primer sequences are listed in the Supplementary Table S1. The average C(t) value of the telomeric repeat reactions was subtracted from the average C(t) value of the control locus reactions to get ΔC(t). Quantification of the telomeric repeats was presented as fold changes relative to telomerase-positive 293T cells or to the untreated condition.

### qRT-PCR

RNA was extracted using RNeasy kit (Qiagen, catalog no. 74004) following the manufacturer's protocol. Extracted RNA was reverse transcribed using the iScript Advanced cDNA synthesis kit (Bio-Rad, catalog no. 1725038). qRT-PCR was performed on the equivalent of 50 ng of cDNA with 500 nmol/L of primer using SsoAdvanced Universal SYBR green Supermix (Bio-Rad, catalog no. 1725271). The following conditions were used for qPCR: 98°C for 30 seconds and 35 cycles at 98°C for 15 seconds and 60°C for 30 seconds. The expression of *TERT*, *TERC*, *GREB1*, and *SPTBN2* were normalized to *HPRT* (Supplementary Table S1). Results were analyzed using CFX Manager Software 3.1 (Bio-Rad) to assess the relative gene expression levels of normalized target genes.

### C-Circle Assay

We performed the ALT-associated C-circle assay following a previously described protocol with some modifications (31). Briefly, DNA was extracted from the cells using DNeasy Blood and Tissue Kit (Qiagen, catalog no. 69506) protocol and then was purified using QiaQuick PCR Purification Kit (Qiagen, catalog no. 28104) following the manufacturer's protocols. Rolling sample amplification reaction mix (containing: 0.2 mg/mL BSA, 0.1% Tween-20, 1X Phi29 reaction buffer, and 1 mmol/L of dGTP, dATP, and dTTP) was added to 150 ng DNA from each sample with or without 2 units of Phi29 polymerase (New England Biolabs, catalog no. M0269L) in 20 µL total reaction volume and incubated at 30°C for 8 hours. The reaction was then inactivated by incubating samples for 20 minutes at 65°C. Reactions were blotted onto positively-charged nylon membrane (Roche) and cross-linked using Stratagene UV Stratalinker (Stratagene). The membrane was washed with 2X saline sodium citrate (2XSCC) buffer with 0.1% SDS, blocked using DIG Easy-Hyb for 1 hour at 42°C (Roche, catalog no. 11093274910). Telomeric repeats were probed by incubating the membrane with telomeric probe containing digoxigenin (5′CCCTAACCCTAACCCTAACCCTAA-DIG; Integrated DNA Technologies; Supplementary Table S1) overnight at 42°C DIG Easy Hyb buffer (Roche). Blots were washed in 2X SSC buffer with 0.1% SDS at room temperature, followed by 0.2X SSC 0.1% SDS at 50°C, and blocked in 5% milk in Tris-buffered Saline with 0.1% Tween-20 (TBST) for 30 minutes at room temperature. Blots were incubated in anti-Digoxigenin-AP, Fab fragments diluted 1:10,000 in the blocking buffer for 30 minutes at room temperature (Roche, catalog no. 11093274910) following the manufacturer's protocols. Blots were washed in 2X SSC buffer with 0.1% SDS at room temperature. C-circle signal was detected using the CDP-Star kit (Roche) according to the manufacturer's instructions and imaged using the ChemiDoc Imaging System (Bio-Rad). U2OS cells (serial dilution from 0–50 ng DNA) were used as an ALT-positive control while PC3 cells (150 ng DNA) were used as a negative control.

### ChIP Analysis for MYCN Binding

Cells were fixed by adding freshly made formaldehyde solution [11% formaldehyde (Sigma catalog no. F8775), 0.1 mol/L NaCl, 1 mmol/L ethylenediaminetetraacetic acid (EDTA), 50 mmol/L HEPES] to the final concentration of 1% formaldehyde directly to the medium. After incubation for 15 minutes at room temperature, formaldehyde was quenched by adding glycine to the final concentration of 0.125 mol/L for 5 minutes at room temperature. The cells were then washed three times in PBS containing 0.5% Igepal and 1X Halt protease inhibitor (Thermo Fisher Scientific, catalog no. 7834). Cells were counted and stored at −80°C until use.

Chromatin shearing and preparation was done on 15 million cells per sample using truChIP Chromatin Shearing Kit with Formaldehyde kit (Covaris, catalog no. 520154) following the manufacturer's protocol. The nuclei were suspended in 1 mL of 1X D3 solution and were sheared using Q800-sonicator (Qsonica) with the following settings: sonicator amplitude setting: 30%, sonication pulse rate: 30 seconds on, 30 seconds off, total sonication on time: 30 minutes, sample process temperature: 4°C. Chromatin size was checked using bioanalyzer (Agilent) and extra sonication was done using the same setting but for 5 minutes increments as needed to get average DNA fragments size between 180 and 350 bps.

Chromatin immunoprecipitation was done using iDeal ChIP-seq kit for Transcription Factors (Diagenode, catalog no. C01010170) following the protocol included the kit. We used 2 µg of mouse anti-MYCN (Santa Cruz Biotechnology, sc-53993, RRID:AB_831602) for immunoprecipitation and we did precipitation in duplicate before combining the beads at the wash steps. Precipitated DNA was suspended in 60 µL DNAse free water and 5 µL of chromatin was used as the input from every sample. CTCF and IgG antibodies (provided in the kit) were used as positive and negative controls.

qPCR was done using 2X SYBR Select master Mix (Thermo Fisher Scientific, catalog no. 4472908). We designed primers that span the two MYCN peaks at the *TERT* promoter. We have also included primers that target MYCN binding sites at *PSAT1* promoter (two primer sets), *ASNS* promoter (two primer sets), and *TXN* promoter (one primer set) as positive MYCN regions. Negative MYCN regions were also included by designing primers that span regions with no MYCN peaks; *ASNS* last exon (two primer sets), and *HPRT* last exon (two primer sets). Primers used in this study are included in the Supplementary Table S2. Samples were run in duplicates and the percentage of enrichment relative to the input was calculated following the Diagenod Kit instructions.

### Bioinformatic Analysis

We mined our previously published data (16), freely available in a web-based server at St. Jude Children's Research Hospital: https://viz.stjude.cloud/st-jude-childrens-research-hospital/visualization/comparison-of-epigenetic-states-in-mycn-amplified-and-atrx-mutated-neuroblastomas∼68.

To generate ChIP-seq data, Burrows-Wheeler Aligner (07.12-r1039, RRID:SCR_010910) was used to align ChIP-seq reads to human genome hg19. Duplicated reads were identified using Picard (RRID: SCR_006525) tools and Samtools (version 1.2, RRID:SCR_002105) and only nonduplicated ones were used in the analysis. At least 10 million reads from H3K4me2/3, H3K9Ac, H3K27Ac, CTCF, RNA, PolymeraseII, BRD4, MYCN and 20 million reads from the rest of the markers per ChIP-seq and input reactions were used. All the used reads had relative strand correlation value >1 as determined using caTools (version 1.17, RRID:SCR_023566) and bitops (version 1.0). MYCN peak calling was done using MACS2 (version 2.1.10, RRID:SCR_013291).

Whole-genome bisulfite data were also aligned to hg19 genome using BSMAP (version 2.74, RRID: SCR_005671). Differentially methylated loci (DML) were called using DMLtest and CallDMR functions in DSS package (RRID:SCR_002754, parameters: *P* threshold for DMR = 0.1, minimum DMR length = 50 bp, minimum CpG sites = 3), as described previously (32, 33).

In this study, we used the previously generated ChromHMM states which were generated using ChromHMM (version 1.10, RRID:SCR_018141) with coalfields 0,1,2,5- center for BinarizeBed as described previously (16, 34). Color intensity was normalized to the maximum total percentage of the state covering a gene including the flanking regions (16). For analyzing HMM states at telomeres, we determined the relative abundance for each HMM state in 5 Mb from chromosomal termini. For genic regions, we included 2-kb upstream and downstream to our analysis.

### Western Blot Analysis

The cells were harvested from a 100-mm tissue culture plate by removing the medium, washing twice with PBS (pH 7.4) and scraping in 1 mL PBS. The cells were then pelleted by centrifugation at 13,000 rpm for 1 minute at 4°C. For histone extraction, cells were resuspended in 250 µL Triton extraction buffer containing 0.5% Triton-X 100, Halt protease inhibitor to a final concentration of 1X (Thermo Fisher Scientific, catalog no. 7834) and EDTA to a final concentration of 5 mmol/L. Cells were incubated in the lysis buffer for 10 minutes on a horizontal shaker at 4°C followed by centrifugation at 6,500 × *g* for 10 minutes at 4°C to collect the nuclei. The supernatant was discarded, and the nuclei were washed with 125 µL of ice-cold Triton extraction buffer. The samples were then centrifuged again 6,500 × *g* for 10 minutes at 4°C and the supernatant was discarded. The pelleted nuclei were resuspended in 100 mL of 0.2N HCl containing 10% glycerol and incubated on a gentle horizontal shaker at 4°C overnight. The debris were pelleted and discarded by centrifuging the tubes at 14,000X for 10 minutes at 4 °C. A total of 90 µL of the supernatant was transferred to a new tube containing 10 µL neutralization buffer containing: 2 mol/L NaOH, 1X Halt protease inhibitor (Thermo Fisher Scientific, catalog no. 7834) and 10 mmol/L Dithiothreitol (DTT). To analyze MYCN and GAPDH using Western Blot, cells were suspended in 1 mL Radioimmunoprecipitation assay (RIPA) Lysis and extraction buffer (Thermo Fisher Scientific, Catalog no. 89900) supplemented with 1X Halt protease inhibitor (Thermo Fisher Scientific, Catalog no. 7834) and incubated at 4 degrees for 15 minutes. Cell lysates were then centrifuged at 15,000 × *g* at 4 degrees for 15 minutes and supernatant was transfered to new tubes. Protein concentration was determined using bicinchoninic acid (BCA) protein assay kit (Thermo Fisher Scientific, catalog no. 232225), following manufacturer's instructions. Equal amount of protein from different samples and 5 µL of Odyssey One-Color Protein Molecular Weight Marker (LI-COR, catalog no. 928-40000) were resolved on 4%–15% SDS-PAGE (Bio-Rad, catalog no. 4561086) then the protein was transferred to a nitrocellulose membrane at 4°C overnight at 30 V. The membrane was washed and blocked for 1 hour at room temperature using Odyssey Blocking buffer, PBS (LI-COR, catalog no. 927-40000). The membranes were probed with Anti-histone H3 Rabbit Ab (Cell Signaling Technology, catalog no. 9715S, RRID:AB_331563), anti-trimethyl Histone H3 (H3K27me3, Cell Signaling Technology, catalog no. 9733S RRID:AB_2616029) diluted 1:1,000, mouse anti-MYCN (1:1000, Santa Cruz Biotechnology, sc-53993, RRID:AB_831602), or rabbit anti-GAPDH (1:2,500, Abcam ab-9485, RRID:AB_307275) for 1 hour at room temperature then washed three times, 5 minutes each with PBS supplemented with 0.1% Tween-20. The membranes then were incubated with IRDye 680CW goat anti-rabbit antibodies (1:5,000, LI-COR, 925-32211, RRID:AB_2651127) or IRDye 800CW goat anti-mouse antibodies (1:5,000, LI-COR, 926-32210, RRID:AB_621842) were used for 1 hour at room temperature followed by washing for three times, 5 minutes each, with PBS supplemented with 0.1% Tween-20. Odyssey CLX infrared gel imaging system (LI-COR Biosciences) was used for scanning the membranes.

### Inducible MYCN Cell Lines

To make the doxycycline-inducible cell lines, cells were transduced with the Lenti-X Tet-One-Puromycine-MYCN lentiviruses as described previously (16). We confirmed the sequence of the construct by Sanger sequencing. Lenti-X Tet-One-Puromycine empty vector (Takara Bio, catalog no. 631847) was used as a control.

### MYCN Short Hairpin RNAs

We purchased three *MYCN*-specific short hairpin RNA (shRNA) SMART lentiviral vectors with Turbo-RFP reporter under hCMV promoter directly from Horizon Discovery as bacterial glycerol stocks [catalog no. V3HSMCR_5948025 (shRNA-A), V3HSMCR_5226249 (#shRNA-B), and V3HSMCR_7110780 (shRNA-C)]. Scambled, nontargeting SMART lentiviral vector with Turbo-RFP reporter was also purchased to serve as a control (catalog no. VSC6571). Bacteria containing the vectors were streaked on ampicillin-LB-agar plate and one colony was selected and grew in 50-mL LB broth containing 100-µg/mL ampicillin overnight. The plasmid was then extracted using ZymoPure Plasmid Midikit (Zymo, catalog no. 4200) following the manufacturer's instructions.

### Lentiviral Preparation and Transduction of Cells to Make Stable Cell Lines

Viral particles were prepared in HEK293T cells by cotransfecting the lentiviral vector containing the inducible MYCN transgene or MYCN-shRNA with the second-generation lentiviral packaging system. In this system, lentiviral genes Gag, Pol, Rev, and Tat are coded on one plasmid, the ENV gene is encoded on the second plasmid while the third plasmid contains the viral Long Terminal Repeats (LTRs) and the packaging signal. A lentiviral vector was mixed with the three packaging plasmids in a ratio of 6:3:1:1, respectively and 15 µg of the mix was used to transfect one 100-mm cell culture plate of approximately 25% confluent HEK293T cells using 30 µL (ratio 1:2) of TransIT-2020 (MirusBio, catalog no. MIR 5404) following the manufacturer's protocol. The supernatants containing the viral particles after 48 and 72 hours were collected, filtered, and concentrated using Lenti-X Concentrator (Takara Bio, catalog no. 631231) following the manufacturer's protocol. The viral titre was determined using qPCR Lentivirus Titer Kit (abm # LV900) following the manufacturer's protocol.

Cells were transduced with Lenti-X Tet-One lentiviruses containing MYCN or with the empty vector at multiplicity of infection of 1 with 8 µg/mL polybrene. Selection for the cells containing the construct was done using puromycin (1–2 µg/mL) and the cells were maintained in puromycin-containing media. Induction of MYCN of MYCN-shRNA was done by adding doxycycline to the media to a final concentration of 0.5 µg/mL.

### Cloning *TERT* Promoter

pGL4.0-*TERT* WT was a gift from Joseph Costello (Addgene plasmid # 84924, RRID: Addgene_84924; ref. 35). This plasmid contains *TERT* core promoter (short construct) upstream to a firefly luciferase construct. We added the *TERT* hypermethylated oncological region (THOR) to the same plasmid to get pGL4.0-*TERT-Long:Luciferse*. To do so, we used PCR reaction to amplify the THOR. The primers contained 20 nucleotides overlaps to the downstream and upstream of *XhoI* and *KpnI* restriction sites of pGL4.0-*TERT* plasmid. Inserting THOR promoter region was done using NEBuilder HiFi DNA Assembly kit (NEB, catalog no. E2621) following the manufacturer's protocol. Sanger sequencing was used to confirm the fidelity of the inserted sequence.

### Luciferase Assay

Cells in 10 cm dishes were transfected with 13.5 µg pGL4.0-*TERT*-short:Luciferse or pGL4.0-TERT-Long:Luciferse and 1.5-µg NanoLuc plasmid (Promega) in 1.5 mL DMEM without serum using 30 µL TranIT2020 (Mirus Bio, catalog no. 5400) following manufacturer's protocol. After 2 days, cells were harvested, and Firefly and Nano luciferase luminescence were measured using Dual Luciferase assay kit (Promega, catalog no. E1910). Firefly luciferase was normalized to NanoLuc readings.

### Data Availability

All the other data supporting the findings of this study are available within the article and its Supplementary Data files and from the corresponding author upon reasonable request.

## Results

### *TERT* Promoter CpGs are Hypomethylated in Neuroblastoma

**FIGURE 1:**
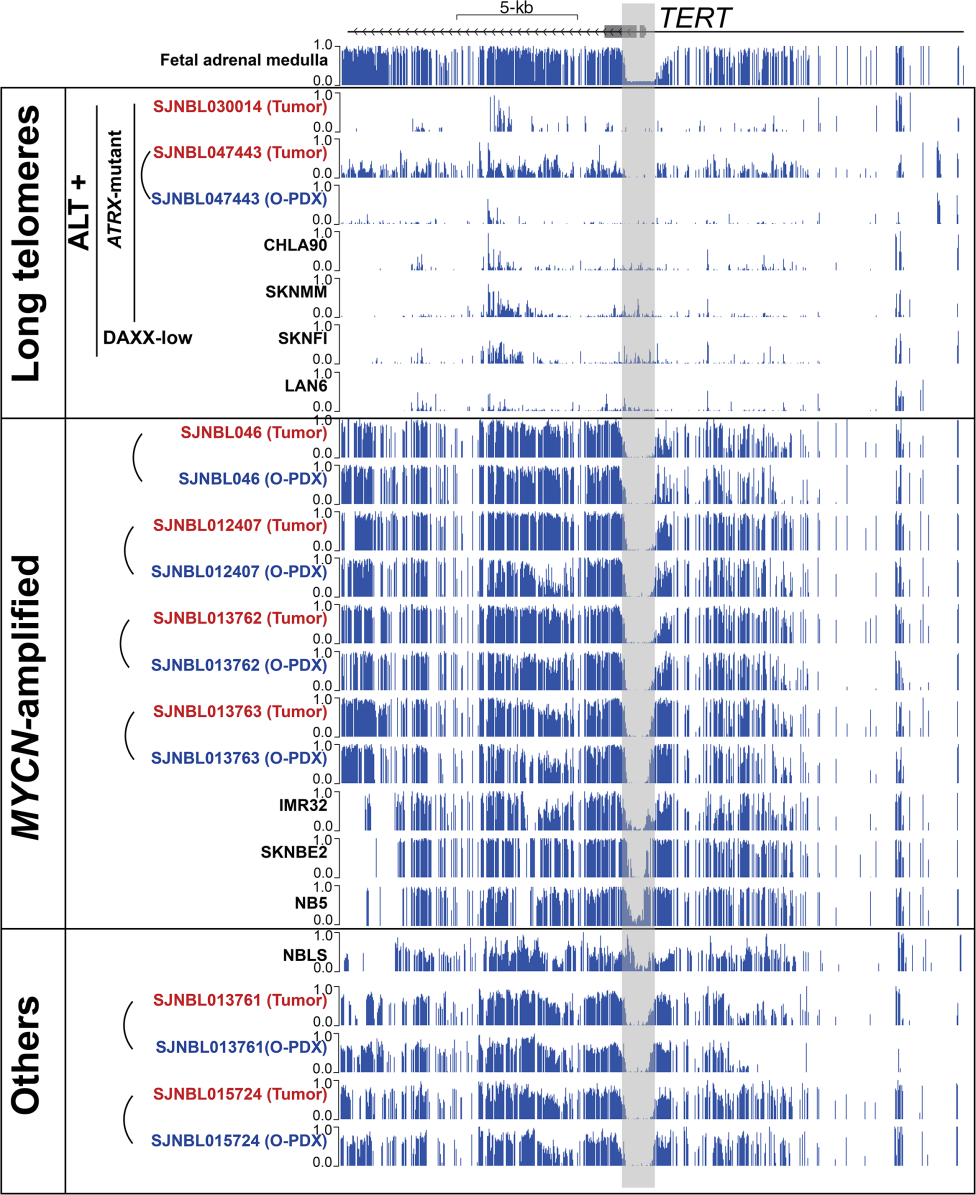
DNA hypomethylation of the *TERT* locus in neuroblastoma cell lines, tumors and O-PDX. **A,** Alignments of DNA methylation from WGBS for the *TERT* locus in neuroblastoma cell lines (black), autopsy (red), and O-PDX (blue). Tumor autopsies and O-PDX that are derived from the same patients are connected by a curved line. The hypomethylated region shaded in gray starts −694 bases from the *TERT* TSS and ends 758 bases after the TSS.

To explore the epigenetic control of *TERT* expression, we leveraged our previously generated WGBS analysis of neuroblastoma (16). In our prior work, we comprehensively characterized the epigenetic marks of eight different cancer autopsies, seven O-PDX derived from these cancer autopsies, as well as eight additional neuroblastoma cell lines. Together, these 23 neuroblastoma samples represent the full spectrum of genetic alterations observed in high-risk neuroblastoma. Here, we analyzed DNA methylation at the *TERT* promoter and found that all neuroblastoma samples contained approximately 1.5 kb of hypomethylated DNA spanning from nucleotides −694 to +758 relative to the *TERT* gene transcription start site (TSS) and overlapping the *TERT* promotor region (Fig. 1). This hypomethylated region surrounding the *TERT* transcriptional start site is also observed in the fetal adrenal medulla (Fig. 1). This is particularly relevant as the fetal adrenal medullae contain progenitors of sympathetic ganglion cells and neuroblastoma is thought to originate from these progenitor cells that have undergone differentiation arrest (36).

### *TERT* Promoter Hypomethylation is Expanded in Neuroblastomas with Long Telomeres

**FIGURE 2:**
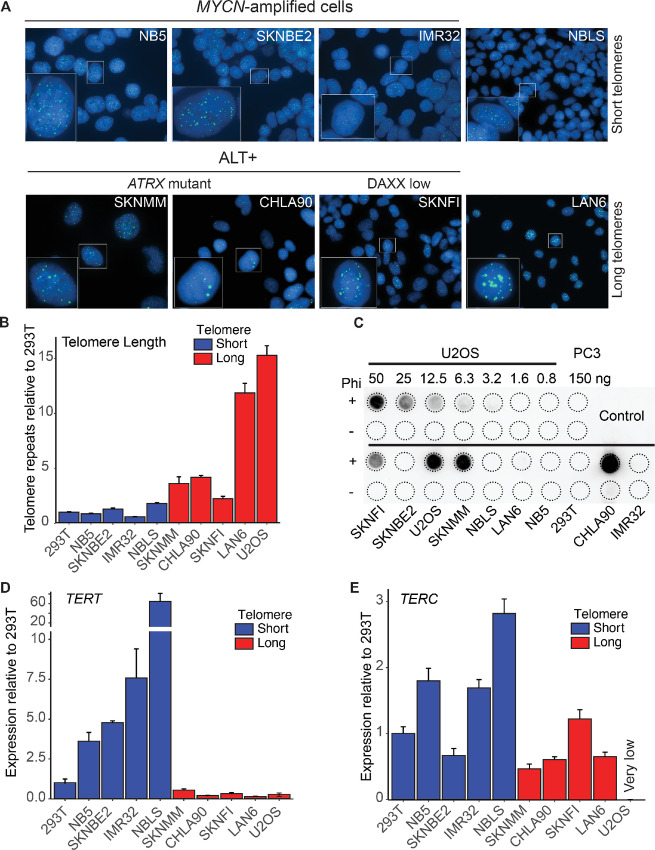
Molecular characterization of telomere maintenance in neuroblastoma cell lines. Telomere maintenance mechanism was assessed in eight neuroblastoma cell lines used in this study: *MYCN*-amplified (NB5, SKNBE2, IMR32), moderate level of MYCN without amplification (NBL-S), *ATRX*-mutant (SKNMM, CHLA90), low-DAXX (SKNFI) and ever-shorter telomere (LAN6) neuroblastoma cells. **A,** Representative images of Telomere FISH (green) showing brighter signal in cell lines with long telomeres. **B,** Telomeric repeats in neuroblastoma cell lines relative to those in the telomerase-positive 293T cells using qPCR. Telomerase-negative, ALT-positive U2OS cells were used as a positive control for long telomeres. **C,** Representative blot of C-circle assay. U2OS cells were used as a positive control and PC3 cells were used as a negative control. The amount of DNA in nanograms (ng) is indicated on the blot. All reactions were done in the presence or absence of the Phi 29 (Phi) DNA polymerase. qRT-PCR was performed in neuroblastoma cell lines to measure *TERT* (**D**) and *TERC* (**E**) expression relative to the telomerase-positive 293T cell line. Gene expression was normalized to *HPRT* expression. The U2OS cell line was used as an ALT-positive control and does not express *TERC*.

The neuroblastoma samples were previously evaluated for *ATRX* and *DAXX* mutations, and *MYCN* amplification. Mutations in the chromatin remodeler *ATRX* and its partner *DAXX* are strongly associated with ALT while *MYCN* amplification is observed in telomerase-dependent/ALT-negative neuroblastomas (16). We confirmed the telomere maintenance mechanisms in the eight neuroblastoma cell lines by performing telomere-specific FISH, telomere qPCR, and the C-circle assay, and measuring the relative expression of the telomerase catalytic subunit *TERT* and the RNA template *TERC* (Fig. 2). Telomere-specific FISH staining revealed ultrabright telomeric foci, consistent with long telomere lengths, in SKNMM, CHLA90, SKNFI, and LAN6, but not in NB5, SKNBE2, IMR32, and NBLS (Fig. 2A). The relative quantity of telomere repeats assessed by real-time qPCR (Fig. 2B) corresponded with the cell lines identified as having relatively longer telomeres. Partially single-stranded extrachromosomal circular DNA containing C-rich telomeric-repeat sequences, called C-circles, are enriched in ALT-positive cancer (37). To measure C-circle levels in our neuroblastoma cell lines, we used a rolling circle amplification-based assay (38). Neuroblastoma cell lines with short telomeres were C-circle negative, while cell lines with long telomeres were positive for C-circles, except for LAN6 (Fig. 2C). *TERT* expression was consistently elevated in neuroblastoma cell lines with short telomeres, while neuroblastoma cell lines with long telomeres had extremely low levels of *TERT* (Fig. 2D). Taken together, these results confirm that SKNMM, CHLA90, and SKNFI are indeed ALT-positive, as they possess the hallmarks characteristic of ALT-positive cancers: long telomeres, very little to no expression of telomerase, and the presence of C-circles. Notably, SKNMM and CHLA-90 carry *ATRX* mutations (16, 39, 40), which are tightly associated with ALT and detected in a significant fraction of high-risk neuroblastoma found in adolescents and young adult patients (16). Although SKNFI has intact ATRX, the expression of DAXX, an established ATRX binding partner and ALT suppressor, is low (16, 41). ALT-associated *DAXX* mutations are observed in pancreatic neuroendocrine tumors but rarely in neuroblastoma (42). Although LAN6 shares many features observed in ALT-positive cancers, the uniformity of telomere lengths, the absence of C-circles, and intact *ATRX* and *DAXX* (16) suggests that it is neither ALT-positive nor telomerase dependent, and may be similar to cancers previously described in the literature as ever-shorter telomeres (6). All neuroblastoma cell lines in this study expressed *TERC*, with long telomere cell lines tending toward lower expression compared with short telomere cell lines (Fig. 2E). Notably, the ALT-positive control cell line U2OS does not express *TERC* (43). We also confirmed *MYCN* amplification status in SKNBE2, IMR32, and NB5 cells using FISH (Supplementary Fig. S1).

When we stratified the neuroblastoma cell lines by short versus long telomeres, a striking pattern emerged when we evaluated the methylation status of the *TERT* promoter CpG island. In neuroblastomas with long telomeres, the hypomethylated region extended beyond the proximal promoter region to include the entire *TERT* locus, and multiple nearby genes (Supplementary Fig. S2). Moreover, the O-PDX models and patient autopsies with *ATRX* mutations confirmed this pattern of extended DNA hypomethylation beyond the *TERT* promoter CpG island (Supplementary Fig. S2). Taken together, these data suggest that telomerase-independent cancer cells with long telomeres are associated with repressed *TERT* expression and hypomethylation of the *TERT* locus in neuroblastoma.

### Neuroblastoma Cells with Long Telomeres are Enriched for Repressive Histone Marks at the *TERT* Locus

**FIGURE 3:**
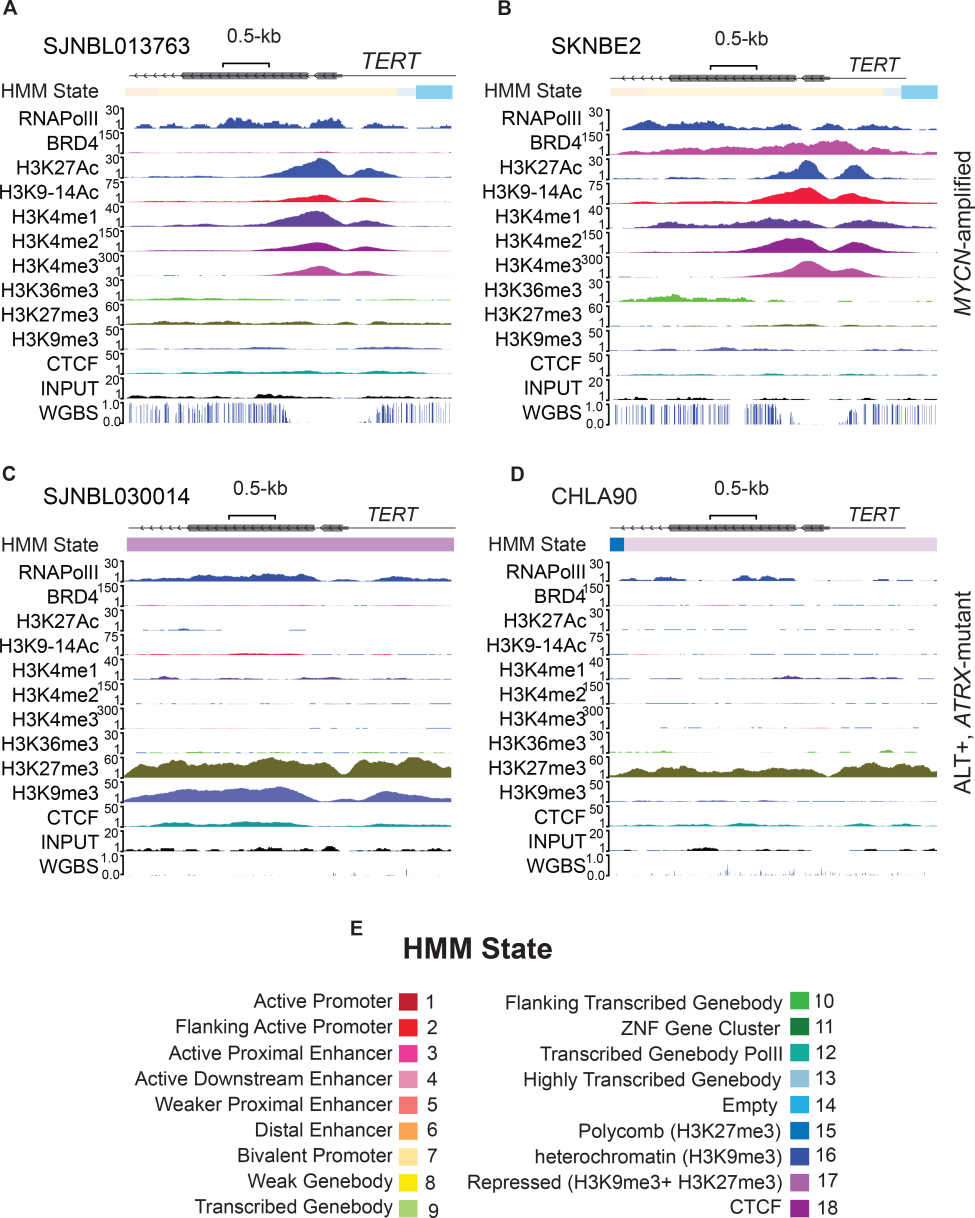
Active chromatin marks in *MYCN*-amplified neuroblastoma and repressive chromatin marks in neuroblastoma cells with long telomeres. ChromHMM states, all ChIP-Seq tracks and WGBS for *MYCN*-amplified O-PDX (SJNBL013763, **A**) and cell line (SKNBE2, **B**) showing active marks in the *TERT* locus. ChromHMM states**,** all ChIP-seq tracks and WGBS for *ATRX*-mutant, ALT-positive O-PDX (SJNBL030014, **C**) and cell line (SKNMM, **D**) showing repressive marks in the *TERT* locus. The color codes for different ChromHMM states are shown in **E**.

Histone tail modifications play key roles in regulating gene expression (23). We examined the chromatin marks at the *TERT* locus and found that neuroblastoma cells with *MYCN* amplification and short telomeres are enriched for chromatin marks indicative of open chromatin including H3K4me3, H3K27Ac, H3K14Ac, RNA PolII, and BRD4 at the *TERT* promoter (Fig. 3A and B). These activating epigenetic marks overlap the region of DNA hypomethylation in the proximal *TERT* promoter, supporting a functional correlation between both the histone and DNA epigenetic marks. Overall, we detected a low level of enrichment for the repressive mark H3K27me3 in the majority of the *MYCN*-amplified neuroblastoma samples, with the exception of the O-PDX sample (SJNBL046) which displayed a relatively high level of H3K27me3 (Supplementary Fig. S3A–S3D). Previously, we employed HMM to bin epigenetic marks into 18 different epigenetic states (ChromHMM; ref. 16). The coexistence of repressive and active marks at the promoter region corresponds to our ChromHMM state 7 (bivalent promoter; ref. 16), which is frequently observed in some genes poised for rapid activation, especially during development (44). Indeed, the proximal *TERT* promoter in fetal adrenal medullary cells showed this bivalent chromatin mark, with the enrichment of both H3K4me3 and H3K27me3 (Supplementary Fig. S3A–S3D). However, because we are unable to ascertain allele-specific epigenetic marks, this bivalent state may also represent monoallelic control of *TERT* expression, with one active allele and another transcriptionally silent allele.

In contrast to the *MYCN*-amplified neuroblastoma cells, the neuroblastoma samples with long telomeres, including cell lines, autopsies and O-PDX tumors, showed repressive chromatin marks, especially H3K27me3 in the same region of the proximal *TERT* promoter (Fig. 3C and D). This H3K27me3 repressive mark overlaps the extended DNA hypomethylated region observed in neuroblastomas with long telomeres including ALT-positive tumors. The *TERT* promoter tended toward ChromHMM 17 state (Fig. 3E), which is characteristic of repressed regions of the genome. Consistent with the active and repressed epigenetic states segregated by short versus long telomeres, *TERT* expression was elevated in cells lines with active chromatin marks and short telomeres compared to cell lines with long telomeres and repressive marks (Fig. 2D).

### *TERT* is a MYCN Target

**FIGURE 4:**
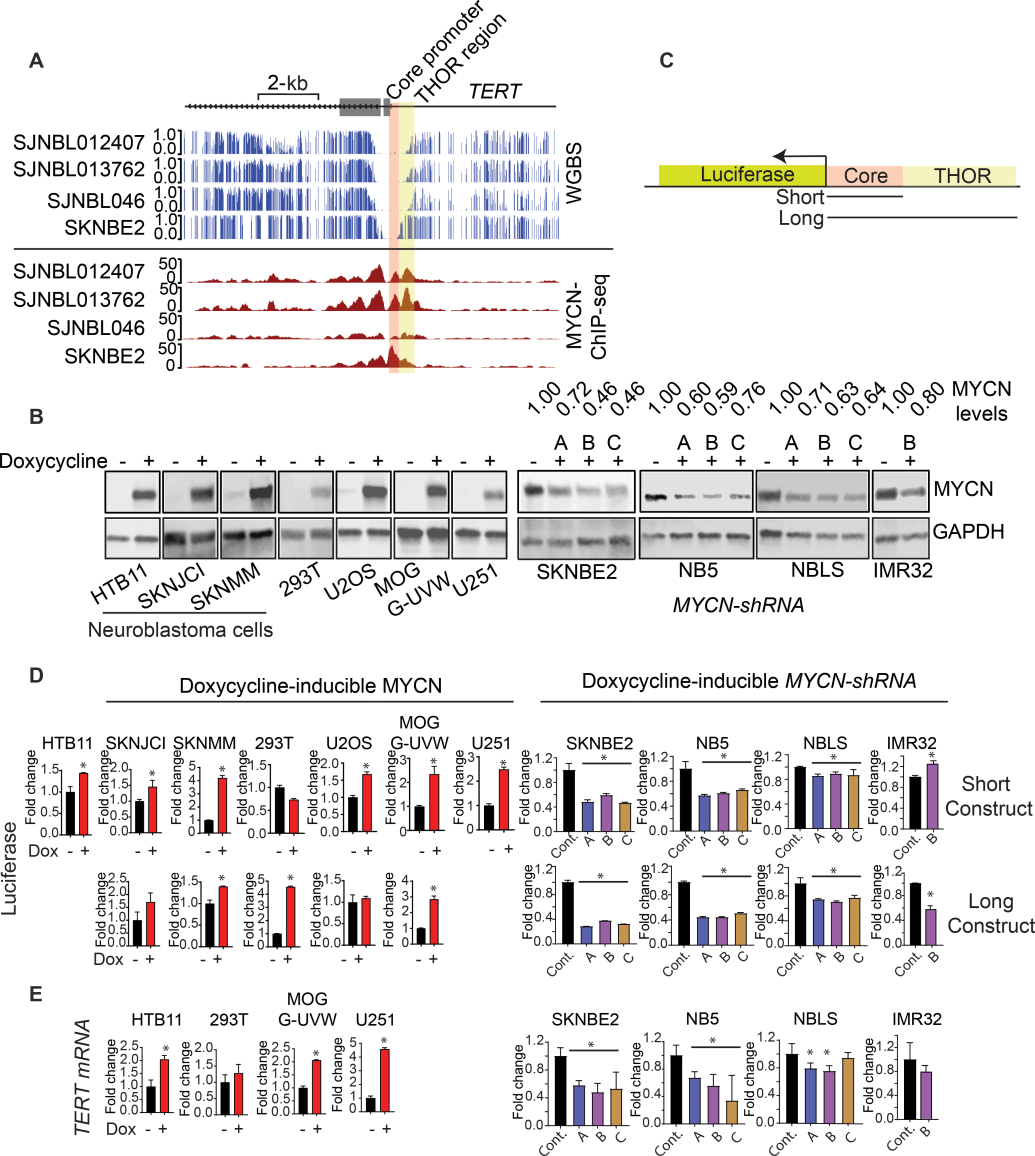
*TERT* is a bona fide MYCN target. **A,** Alignments of DNA methylation from WGBS and MYCN-ChIP-seq in three *MYCN*-amplified neuroblastoma O-PDX models and cell line (SKNBE2) showing that MYCN binding overlaps with the hypomethylated region in the *TERT* promoter. The core promoter is shaded in red and THOR is shaded in yellow. **B,** Western blot analysis for MYCN and GAPDH (loading control) in different indicated doxycycline-inducible engineered cell lines to ectopically express MYCN in non–*MYCN*-amplified cells (left) or to express three different *MYCN-shRNAs* (indicated as A, B, C) in *MYCN*-amplified neuroblastoma cells (right). Relative MYCN band intensities in cells expressing *MYCN-shRNA* are indicated on top of the blots. **C,** A schematic representation of Luciferase reporter construct. The long construct contains the luciferase gene, and the *TERT* promoter core and THOR sequences, while the short construct does not contain the THOR promoter sequence. **D,** Fold change of Luciferase luminescence for the short construct (top) and the long construct (bottom) compared with control vehicle-treated controls. **E,** Fold change of *TERT* expression in the same cells measured by qRT-PCR. *, *P* < 0.05, error bars represent SD.

Next, we wanted to study the control of *TERT* expression in *MYCN*-amplified neuroblastoma cells. It was previously shown that the *TERT* proximal promoter is comprised of a core promoter located around the TSS (from +51 to −217), and a hypermethylated 433-bp region immediately upstream to the core promoter referred to as the THOR (17, 45). As shown in Fig. 4A, the DNA hypomethylated region surrounding the TSS of *TERT* in neuroblastoma cells overlaps with this previously described core promoter. Moreover, in *MYCN*-amplified neuroblastoma, we observed that MYCN occupies this same hypomethylated region in our MYCN-ChIP-seq analysis (Fig. 4A). Together, these data suggest that *TERT* is a MYCN target and the *TERT* promoter hypomethylated region in neuroblastoma may play a role in the regulation of *TERT* expression.

To confirm the role of MYCN-mediated *TERT* expression, we engineered seven non–*MYCN*-amplified cell lines to ectopically express MYCN under a doxycycline inducible promoter (Fig. 4B). Three of these lines are neuroblastoma cells (HTB-11, SKNJCI, and SKNMM), two are glioblastoma cells (MOG-U-UVW and U251) and one was the transformed human embryonic kidney cell line 293T. In addition, we genetically modified four *MYCN*-amplified neuroblastoma cells (SKNBE2, NB5, NBLS, and IMR-32) to express three different doxycycline-inducible *shRNA* that knock downed MYCN protein levels (Fig. 4B). To monitor MYCN-mediated expression as a consequence of *TERT* promoter binding, we used two luciferase reporter constructs: one with the *TERT* core promoter (short construct) and another one that spans both the core promoter and THOR (long construct; Fig. 4C). Notably, these promoter regions correspond to two MYCN binding peaks in *MYCN*-amplified neuroblastoma cells (Fig. 4A). By generating the two reporter constructs, we could assess whether binding to the THOR sequences in addition to the core promoter sequence impacts *TERT* expression. We transfected the genetically modified cell lines with the reporter constructs and doxycycline was used to ectopically express either *MYCN* or *MYCN-shRNA* transgenes. We found that inducing MYCN in non–*MYCN*-amplified cells increased the luciferase expression in these cells. Conversely, knocking down MYCN by inducing *MYCN-shRNA* in *MYCN*-amplified cells reduced luciferase expression in these cells (Fig. 4D). Moreover, the response was more pronounced with the long construct (containing both the *TERT* core promoter and THOR, Fig. 4D bottom) relative to the short construct (containing the core promoter only, Fig. 4D top).

We also measured the expression of the endogenous *TERT* following modulating MYCN levels using doxycycline-inducible transgenes. Consistent with our luciferase data, we found that induction of MYCN increased expression of *TERT* in HTB-11, MOG-U-UVW, and U251 cells (Fig. 4E). Notably, two neuroblastoma cell lines with long telomeres SKNMM and SKNJCI did not show increase in *TERT* expression upon induction of MYCN. The repressed *TERT* locus observed in SKNMM (Fig. 3) suggested that MYCN does not bind to the *TERT* promoter in these cells. Indeed, MYCN ChIP-seq analysis shows the absence of MYCN occupancy in the *TERT* promoter in SKNMM cells (Supplementary Fig. S4A). Interestingly, SKNJCI has also very low level of *TERT* expression. This made us expect that SKNJCI cells have long telomeres, which we tested and found this is the case (Supplementary Fig. S4B). Collectively, these data confirm that *TERT* is a bona fide MYCN target and heterochromatic repression of *TERT* prevents MYCN access to induce *TERT* expression.

### Long Telomeres are Associated with Repressed Subtelomeric Regions

**FIGURE 5:**
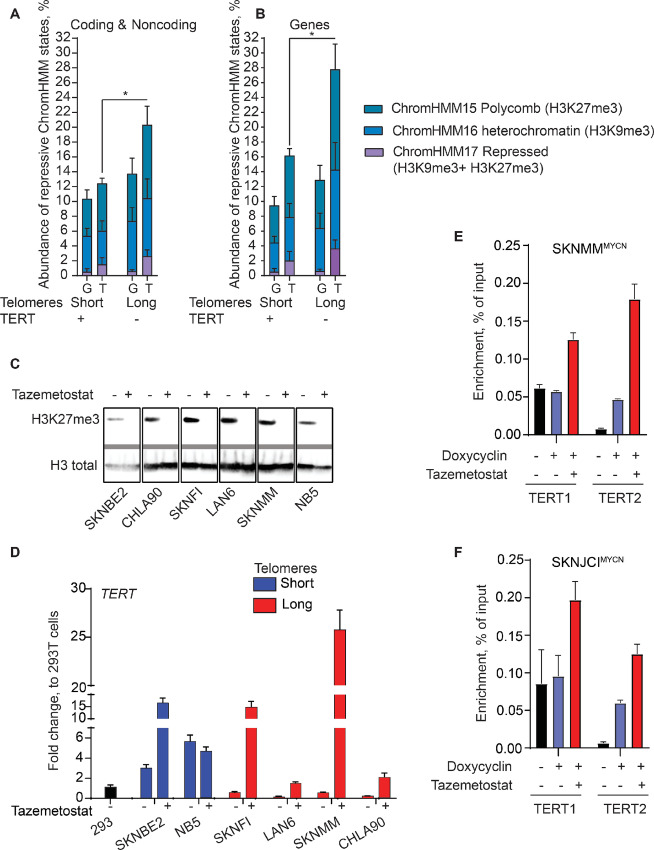
TPE suppresses genes at chromosomal ends of neuroblastoma cells with long telomeres. The average of the percentage of repressive chromatin ChromHMM states at chromosomal ends, subtelomeric region referred to as “T” and genome-wide referred to as “G” for all coding and noncoding sequences (**A**) and genic regions (**B**) in neuroblastoma cells with short and long telomeres. **C** and **D,** Pharmacologic inhibition of EZH2 induces *TERT* expression in *TERT*-negative neuroblastoma cells. **C,** Western blot analysis for H3K27me3 and H3K27 total in neuroblastoma cells after treatment with tazemetostat or the drug vehicle only. **D**, Fold change of *TERT* expression in the same cells after the same treatment measured by qRT-PCR. **E** and **F,** Inhibiting EZH2 facilitates MYCN binding to the *TERT* promoter in neuroblastoma cells with long telomeres. ChIP-qPCR enrichment of MYCN at the *TERT* promoter in SKNMM^MYCN^ (E) and SKNJCI^MYCN^ (F) after treating the cells with tazemetostat for 3 weeks and doxycycline induction of MYCN for 2 days. As *MYCN* is not expressed in both cell lines, MYCN enrichment in the absence of doxycycline represents background. *, *P* < 0.05, error bars represent SD.

Long telomeres may loop back over chromosomal ends, interacting with many genes over long distances and repressing their expression (46–48). Because *TERT* is close to the chromosome 5p terminus, it suggests that this looping may constitute a feedback mechanism to repress *TERT* expression in cells with long telomeres, a phenomenon termed telomere position effect (TPE; refs. 46–48). We explored a possible TPE in neuroblastoma cells with long telomeres and quantified the abundance of chromatin in repressive states 5 Mb from chromosomal ends and performed a similar analysis genome-wide to verify that changes at chromosomal ends do not represent genome-wide differences in repressive marks among these cells. We focused on the three repressive states: ChromHMM 15 (polycomb repressed, enriched for H3K27me3), ChromHMM 16 (heterochromatin, enriched for H3K9me3), and ChromHMM 17 (repressed, enriched for both H3K9me3 and H3K27me3). We found that, relative to the whole genome, repressive marks are more abundant near chromosomal ends in all neuroblastoma cells. However, chromosomal termini from neuroblastoma cells with long telomeres are enriched for repressive marks relative to those from short telomere/telomerase-positive neuroblastoma cells (Fig. 5A; Supplementary Fig. S5A–S5C; Supplementary Table S3A–S3D). In addition, the majority of *TERT*-positive neuroblastoma cells are *MYCN*-amplified. This suggests that the enrichment of repressive marks at chromosomal ends in cells with long telomeres relative to those with short telomeres may represent a reduction in repressive marks due to *MYCN* amplification rather than TPE. To test this possibility, we induced *MYCN* expression in a neuroblastoma cell line with long telomeres that has a doxycycline inducible *MYCN* transgene, SKNMM^MYCN^ (Fig. 4). We found no significant change in the abundance of the repressive ChromHMM states at chromosomal ends in this cell line upon *MYCN* induction (Supplementary Fig. S5C). Taken together, these data suggest that long telomeres are associated with repressive chromatins at the chromosomal ends in neuroblastoma cells.

It was previously shown that TPE represses genes at long distances from chromosomal ends (46–48). To test whether long telomeres have differential repressive effects on genes at chromosomal ends, we further analyzed chromatin repressive marks at chromosomal ends in genic (genes + 2 kb upstream and downstream) regions. Again, repressive marks were more abundant at genes located near the chromosomal ends relative to whole genome in all neuroblastoma cells. However, this enrichment was more evident in neuroblastoma cells with long telomeres (Fig. 5B; Supplementary Fig. S5A–S5C; Supplementary Tables S3A–S3D). *MYCN* induction in SKNMM^MYCN^ cells did not significantly alter this enrichment (Supplementary Fig. S5C). These data suggest that TPE is detected in neuroblastoma cells with long telomeres and TPE may also play a role in repressing *TERT* expression.

### Inhibition of EZH2 Induces TERT Expression in Neuroblastoma Cells with Long Telomeres

EZH2 is the enzymatically active subunit of the PRC2, methylating lysine 27 on histone H3 tails to repress gene transcription (23). To examine the impact of H3K27me3 in the transcriptional silencing of the *TERT* locus in neuroblastoma, we treated neuroblastoma cell lines with long telomeres (SKNFI, SKNMM, CHLA-90, and LAN6) and *MYCN*-amplified neuroblastoma cell lines with short telomeres (SKNBE2 and NB5) with the FDA-approved EZH2 inhibitor, Tazemetostat, and measured *TERT* expression. We treated neuroblastoma cell lines with a range of tazemetostat doses (0–20 µmol/L) and at two timepoints (10 and 21 days) and found treating cells with 20 µmol/L of tazemetostat for 21 days reduced methylated H3K27 below the limit of detection as assessed by Western blot analysis (Fig. 5C). Following 21 days of treatment with 20 µmol/L of tazemetostat, we observed an induction in *GREB1* and *SPTBN2* (Supplementary Fig. S6A and S6B), genes previously shown to be transcriptionally repressed by H3K27me3 in neuroblastoma cells (39). Interestingly, we also observed that inhibiting EZH2 derepressed *TERT* and induced expression in neuroblastoma cells with long telomeres, relative to the housekeeping gene *HPRT* (Fig. 5D; Supplementary Fig. S6C). Moreover, treating *TERT*-negative neuroblastoma cells with two other EZH2 inhibitors (UNC1999 and PF-06821497 acetate) induced the expression of *TERT* (Supplementary Fig. S7A and S7B), confirming the role of PCR2 complex in repressing *TERT* locus in neuroblastoma cells with long telomeres. However, the activation of *TERT* expression was not accompanied by changes in C-circle levels, a canonical ALT-associated hallmark (Supplementary Fig. S7C). The enrichment of H3K27me3 at the *TERT* proximal promoter in neuroblastoma cells with long telomeres, and the induction of *TERT* expression following EZH2 inhibition in those cell lines, support the notion that PRC2 plays an important role in repressing telomerase in telomerase-independent neuroblastoma.

### Inhibition of EZH2 Induces MYCN Binding to the TERT Promoter in Neuroblastoma Cells with Long Telomeres

As inhibiting EZH2 led to MYCN-induced *TERT* expression in neuroblastoma cells with long telomeres, we wondered whether inhibiting EZH2 facilitates MYCN binding to the *TERT* promoter. To address this, we used tazemetostat for 3 weeks to inhibit EZH2 in two neuroblastoma cell lines with long telomeres containing doxycycline-inducible *MYCN* transgene (SKNMM^MYCN^ and SKNJCI^MYCN^). Doxycycline was then used to induce MYCN expression for 2 days before harvesting these cells. Using chromatin immunoprecipitation followed by qPCR (ChIP-qPCR), we assessed MYCN binding to the *TERT* promoter. Our analysis also included two *MYCN*-amplified neuroblastoma cell lines (SKNBE2 and NB5), and three other cell lines with doxycycline-inducible *MYCN* transgene; HTB11^MYCN^ (*MYCN*-nonamplified neuroblastoma cell line with short telomere) and U251^MYCN^ and MOG-U-UVW^MYCN^ (*MYCN*-non-amplified/non-neuroblastoma cells). For each sample, we used two sets of primers targeting MYCN binding sites identified by MYCN-ChIP-seq (Fig. 4A) at the *TERT* promoter. In addition, we also included primers that span MYCN binding sites in the genome: *PSAT1* promoter (two primer sets), *ASNS* promoter (two primer sets), and *TXN* promoter (one primer set) as positive MYCN regions (Supplementary Fig. S8A–S8C). Moreover, primers that span *ASNS* last exon and *HPRT* last exon regions (no MYCN peaks in MYCN-ChIP-seq) were also used (Supplementary Fig. S8C and S8D).

Our findings reveal consistent MYCN (endogenous and induced) binding to the previously identified positive regions with minimal or no enrichment at the negative control sites (Supplementary Table S4A–S4D). Notably, MYCN was enriched at the *TERT* promoter in *MYCN*-amplified neuroblastoma cells (SKNBE2, NB5). Inducing MYCN also led to increased MYCN binding to the *TERT* promoter in HTB11^MYCN^, U251^MYCN^, and MOG-U-UVW^MYCN^ (Supplementary Table S4A–S4D). In neuroblastoma cells with long telomeres (SKNMM^MYCN^ SKNJCI^MYCN^), induction of MYCN resulted in modest or no increase in MYCN enrichment at the *TERT* promoter. However, inhibiting EZH2 by tazemetostat increased MYCN binding to the *TERT* promoter (Fig. 5E and F; Supplementary Table S4). Overall, these findings suggest that inhibiting EZH2 facilitates MYCN binding to the *TERT* promoter, consequently inducing *TERT* expression.

## Discussion

The epigenetic control of *TERT* expression is not well understood (29). Here, we leveraged our extensive neuroblastoma epigenetic database (16) to understand how the *TERT* promoter is regulated in neuroblastoma. The *TERT* proximal promoter, spanning nucleotides +51 to −650 from the TSS, is composed of a core promoter and an upstream region that has been previously described as the THOR (17, 45). Several transcriptional factors bind to the core promoter (17). Indeed, we have shown that MYCN binds to the *TERT* promoter and induces the expression of the gene. Broadly, cancer cells and immortalized cell lines are hypermethylated at CpGs in the core *TERT* promoter and THOR (27), including cancers of the central nervous system (49). However, we show that neuroblastoma cells are exceptional, displaying CpG hypomethylation within the core promoter and THOR, similar to stem cells and nonmalignant somatic cells (27, 49). Notably, the developing adrenal medullary cells show a similar CpG hypomethylation at *TERT* promoter DNA. Taken together, these data are consistent with the idea that most neuroblastoma tumors arise from sympathetic progenitor cells arrested during development (36).

The role of *TERT* promoter DNA methylation in the regulation of *TERT* expression is not well established (29, 50). Generally, CpG methylation is considered a repressive mark, blocking the binding of transcription factors to promoters (23). Paradoxically, *TERT* expression is associated with promoter DNA hypermethylation (50). While it has been suggested that *TERT* promoter DNA methylation prevents CTCF from binding and repressing *TERT* expression (51), several studies have shown that monoallelic *TERT* expression preferentially comes from alleles with hypomethylated promoters (27, 28, 52, 53). This *TERT* promoter CpG hypermethylation phenomenon may be attributed as an attempt by cancer cells to sustain only the minimal level of telomerase needed for telomere length maintenance (28). This hypothesis is supported by the fact that *TERT* amplification is relatively rare in cancer and most telomerase-positive cancer cells have short telomeres (2, 7).

To gain a more comprehensive appreciation of the epigenetic regulation of the *TERT* proximal promoter, we complemented our CpG methylation data with extensive chromatin analysis. Using MYCN ChIP-seq data as well as qRT-PCR and reporter assays, we showed that *TERT* is a bona fide MYCN target in neuroblastoma cells. These data confirm previous observations that overexpressing MYCN is associated with induction of *TERT* expression while downregulating MYCN results in reduced *TERT* mRNA level (11). The *TERT* promoter in telomerase-positive neuroblastoma cell lines showed enrichment for both the repressive mark H3K27me3 and the active mark H3K4me3. While this combination of repressive and active marks has been observed in other regulated genes and described as a bivalent promoter (44), it is also possible that these marks do not coexist on the same allele. Indeed, previous studies have shown monoallelic *TERT* expression (27, 52).

In addition to telomerase-dependent neuroblastoma cells, a subset of high-risk neuroblastoma cells is telomerase independent. While neuroblastoma cells harboring *ATRX* mutations are thought to rely on ALT to maintain telomere lengths (18), a recent study has shown that neuroblastoma tumors can also be negative for both ALT and telomerase. These tumors are characterized by very long telomeres (>20 kbps) that allow these cells to pass through many replication cycles without inducing senescence and apoptosis (6). We found that neuroblastoma cells with long telomeres, with little to no telomerase expression, possess a unique epigenetic signature. Strikingly, neuroblastomas with long telomeres display DNA CpG hypomethylation and enrichment of the chromatin repressive mark H3K27me3 that extends for tens of kilobases around the *TERT* locus and includes neighboring genes. Notably, our findings show that inhibiting EZH2 (the enzymatically active subunit of PRC2) induces *TERT* expression and facilitates MYCN binding to the *TERT* promoter in neuroblastoma cells with long telomeres, suggesting that H3K27 trimethylation is the underlying mechanism responsible for silencing *TERT* in these cells. How PRC2 complex is targeted to the *TERT* locus is not known and the mechanism of DNA hypomethylation is also not clear. Previous work by others has shown a similar signature of H3K27 trimethylation and DNA hypomethylation (termed DNA methylation valleys) in some developmentally repressed genes (54). Importantly, DNA demethylases (TET enzymes) were shown to induce DNA hypomethylation in these valleys (54). In addition, similar to *TERT* in most cancer types, activation of genes in DNA methylation valleys is associated with DNA hypermethylation (54). Taken together, these data suggest that the *TERT* locus is a part of a DNA methylation valley that is epigenetically controlled during development.

MYCN is normally expressed during the development of sympathetic ganglion cells (36). Neuroblastoma arises from developing sympathetic ganglion cells that underwent differentiation arrest (36). Indeed, similar to that in developing sympathetic ganglion cells, the *TERT* promoter in *MYCN*-amplified neuroblastoma cells shows active chromatin marks and DNA methylation of the *TERT* gene body. On the other hand, neuroblastoma cells with long telomeres, including ALT-positive tumors, show a different epigenetic signature at the *TERT* promoter. This different epigenetic pattern may suggest that neuroblastoma with long telomeres may arise from sympathetic progenitor cells at a developmental stage different from telomerase-dependent neuroblastomas in which the *TERT* locus is silenced by PRC2 in a DNA methylation valley. However, characterizing the *TERT* epigenetic signature of sympathetic neuron progenitor cells at different developmental stages are required to test this hypothesis, which is beyond the scope of this study.

Previous work has shown DNA hypomethylation at subtelomic regions in ALT-positive non-neuroblastoma cells (55, 56). Here, we showed enrichment of chromatin repressive marks close to chromosomal ends relative to whole genome. While others have reported widespread DNA hypomethylation in ALT-positive cells (55), our data demonstrated that the repressive chromatin marks are enriched close to chromosomal ends and are more evident at genic rather than intergenic regions. Cells with long telomeres had more abundant repressive marks and repressive states relative to telomerase-positive neuroblastoma cells suggesting a TPE. Notably, TPE was previously described as a negative feedback mechanism to inhibit *TERT* expression in cells with long telomeres (46, 47), and *TERT* is close to the chromosome 5p end. However, this observation does not explain the repression of other genes in the region. An intriguing idea is that repression of subtelomeric genes in cells with long telomeres may work as a barrier to acquire and maintain long telomeres. In this model, cells with long telomeres, for example ALT-positive cells, must find a way to compensate for the repression of genes at chromosomal ends. TPE may also explain the relatively stable short telomeres detected in most telomerase positive cancer cells (46). Minimal telomerase expression will balance the positive selection for continual cellular proliferation without going through telomere attrition-inducing senescence and a potential negative selection of suppressing the expression of subtelomeric genes. Future studies are needed to explore these possibilities.

In conclusion, we show that the *TERT* region is differentially regulated in neuroblastoma cells depending on the mechanism of telomere maintenance. In addition, our data demonstrate that *TERT* promoter DNA methylation is unique in neuroblastoma, with a CpG methylation pattern similar to somatic and stem cells, supporting the hypothesis that neuroblastoma represents an arrested differentiation of sympathetic neuron progenitors. Overall, these findings reveal new insights in the epigenetic regulation of the *TERT* promoter in neuroblastoma. Notably, in terms of treatment, the utility of EZH2 inhibitors in neuroblastoma may in part be influenced by telomere maintenance mechanism.

## Supplementary Material

Supplementary Figure S1 Supplementary Figure S1

Supplementary Figure S2 Supplementary Figure S2

Supplementary Figure S3 Supplementary Figure S3

Supplementary Figure S4 Supplementary Figure S4

Supplementary Figure S5 Supplementary Figure S5

Supplementary Figure S6 Supplementary Figure S6

Supplementary Figure S7 Supplementary Figure S7

Supplementary Figure S8 Supplementary Figure S8

Supplementary tables Supplementary tables 1-4
