# Supplementary material for: The TERT Promoter is Polycomb-Repressed in Neuroblastoma Cells with Long Telomeres
Source: Cancer Res Commun. 2024 Jun 20;4(6):1533–47. doi: 10.1158/2767-9764.CRC-22-0287 (PMC11188873; doi:10.1158/2767-9764.CRC-22-0287)
Supplement: Supplementary Figure S1 [file crc-22-0287-s01.pdf]

Figure S1

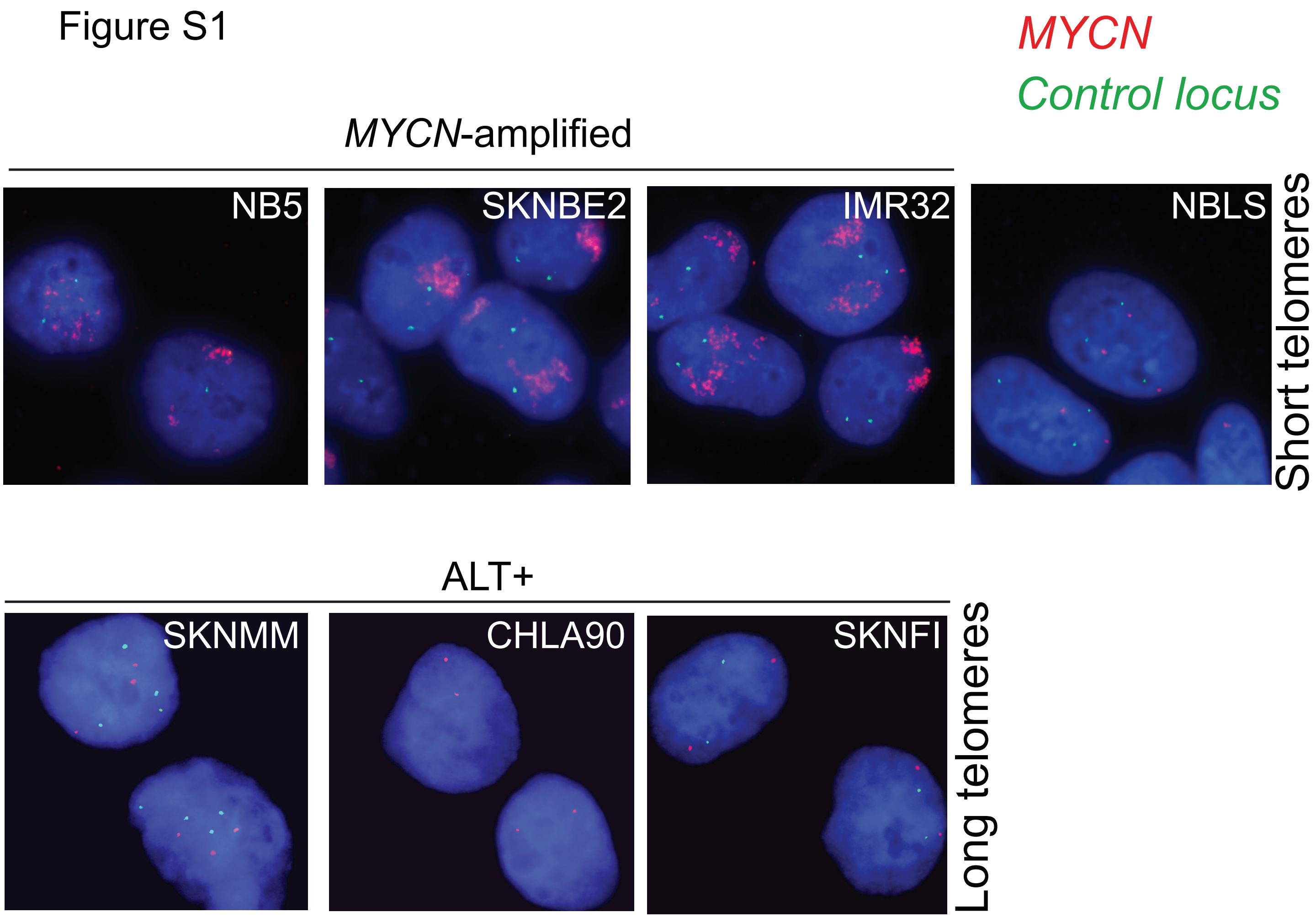

Supplementary figure S1: Representative fluorescence microscopy images of *MYCN*-FISH (red) and a control locus (green) of seven neuroblastoma cell lines used in this study.
