# Supplementary material for: The TERT Promoter is Polycomb-Repressed in Neuroblastoma Cells with Long Telomeres
Source: Cancer Res Commun. 2024 Jun 20;4(6):1533–47. doi: 10.1158/2767-9764.CRC-22-0287 (PMC11188873; doi:10.1158/2767-9764.CRC-22-0287)
Supplement: Supplementary Figure S2 [file crc-22-0287-s02.pdf]

Figure S2

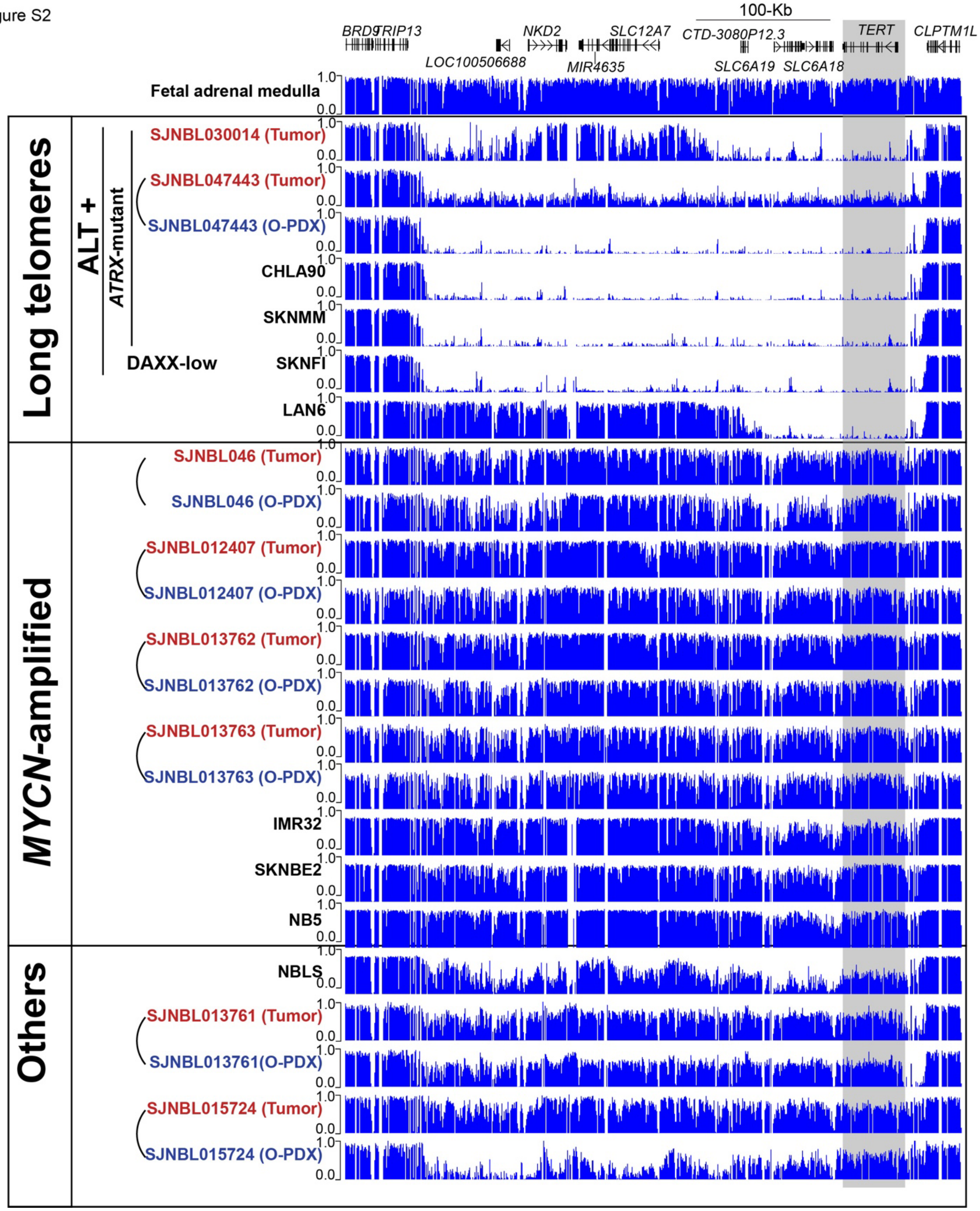

Supplementary figure S2: The DNA hypomethylated region extends for hundereds of kilobases in neuroblastoma cell cells with long telomeres. *TERT* locus is shaded in grey.
