# Supplementary material for: The TERT Promoter is Polycomb-Repressed in Neuroblastoma Cells with Long Telomeres
Source: Cancer Res Commun. 2024 Jun 20;4(6):1533–47. doi: 10.1158/2767-9764.CRC-22-0287 (PMC11188873; doi:10.1158/2767-9764.CRC-22-0287)
Supplement: Supplementary Figure S3 [file crc-22-0287-s03.pdf]

Figure S3

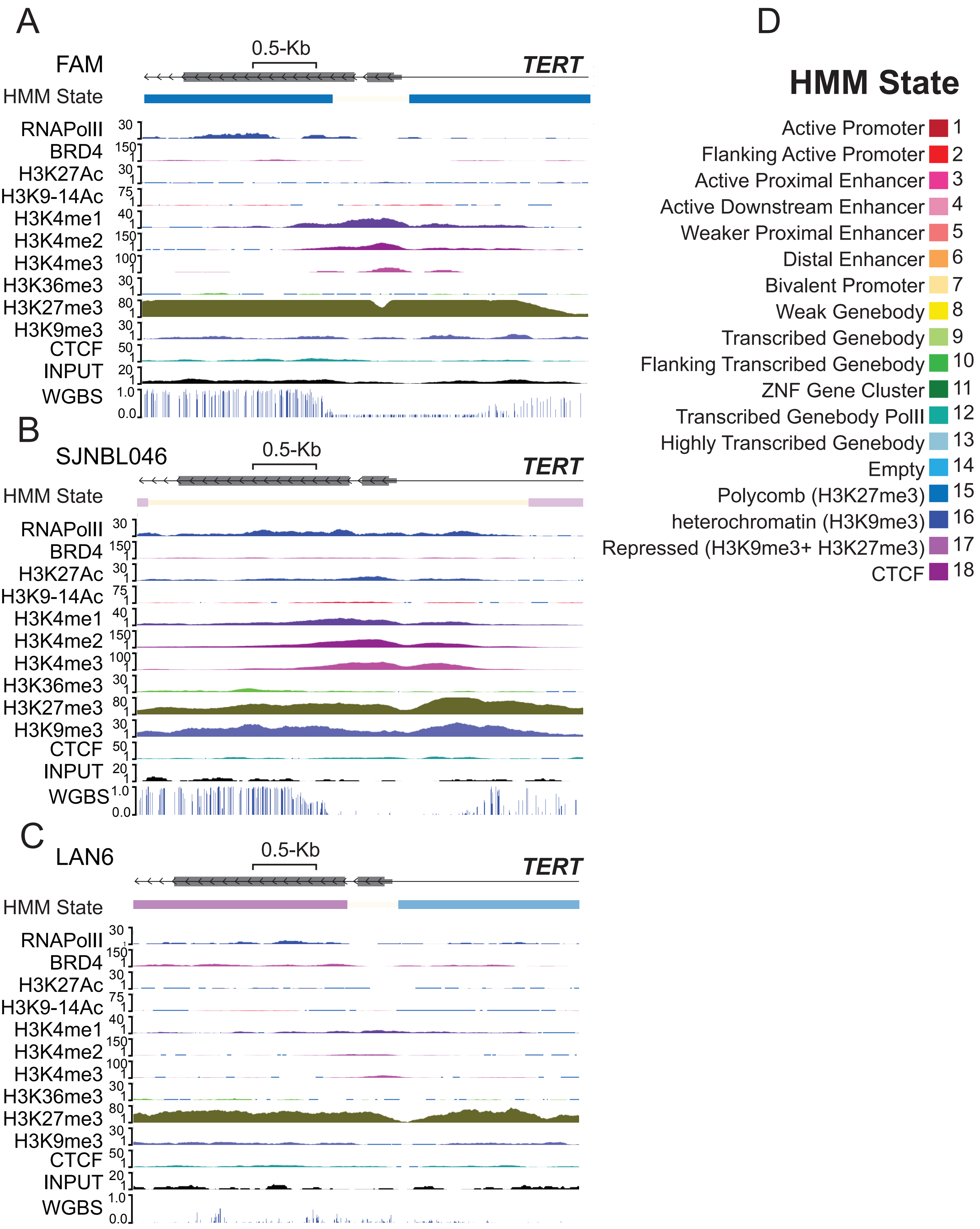

A
FAM
0.5-Kb
TERT
HMM State
RNAPolII
BRD4
H3K27Ac
H3K9-14Ac
H3K4me1
H3K4me2
H3K4me3
H3K36me3
H3K27me3
H3K9me3
CTCF
INPUT
WGBS
B
SJNBL046
0.5-Kb
TERT
HMM State
RNAPolII
BRD4
H3K27Ac
H3K9-14Ac
H3K4me1
H3K4me2
H3K4me3
H3K36me3
H3K27me3
H3K9me3
CTCF
INPUT
WGBS
C
LAN6
0.5-Kb
TERT
HMM State
RNAPolII
BRD4
H3K27Ac
H3K9-14Ac
H3K4me1
H3K4me2
H3K4me3
H3K36me3
H3K27me3
H3K9me3
CTCF
INPUT
WGBS
D
HMM State
Active Promoter 1
Flanking Active Promoter 2
Active Proximal Enhancer 3
Active Downstream Enhancer 4
Weaker Proximal Enhancer 5
Distal Enhancer 6
Bivalent Promoter 7
Weak Genebody 8
Transcribed Genebody 9
Flanking Transcribed Genebody 10
ZNF Gene Cluster 11
Transcribed Genebody PolII 12
Highly Transcribed Genebody 13
Empty 14
Polycomb (H3K27me3) 15
heterochromatin (H3K9me3) 16
Repressed (H3K9me3+ H3K27me3) 17
CTCF 18

Supplementary figure S3: ChromHMM state, all ChIP-Seq tracks and whole geneome bisulfite sequencing (WGBS) for neuroblastoma and fetal adrenal medulla (FAM). **A**) Fetal adrenal medalla. **B)** *MYCN*-amplified O-PDX, SJNLB046. **C**) The neuroblastoma cell line with ever-shorter telomeres, LAN6. **D)** The color codes for different ChromHMM states**.**
