# Supplementary material for: The TERT Promoter is Polycomb-Repressed in Neuroblastoma Cells with Long Telomeres
Source: Cancer Res Commun. 2024 Jun 20;4(6):1533–47. doi: 10.1158/2767-9764.CRC-22-0287 (PMC11188873; doi:10.1158/2767-9764.CRC-22-0287)
Supplement: Supplementary Figure S4 [file crc-22-0287-s04.pdf]

Figure S4

A

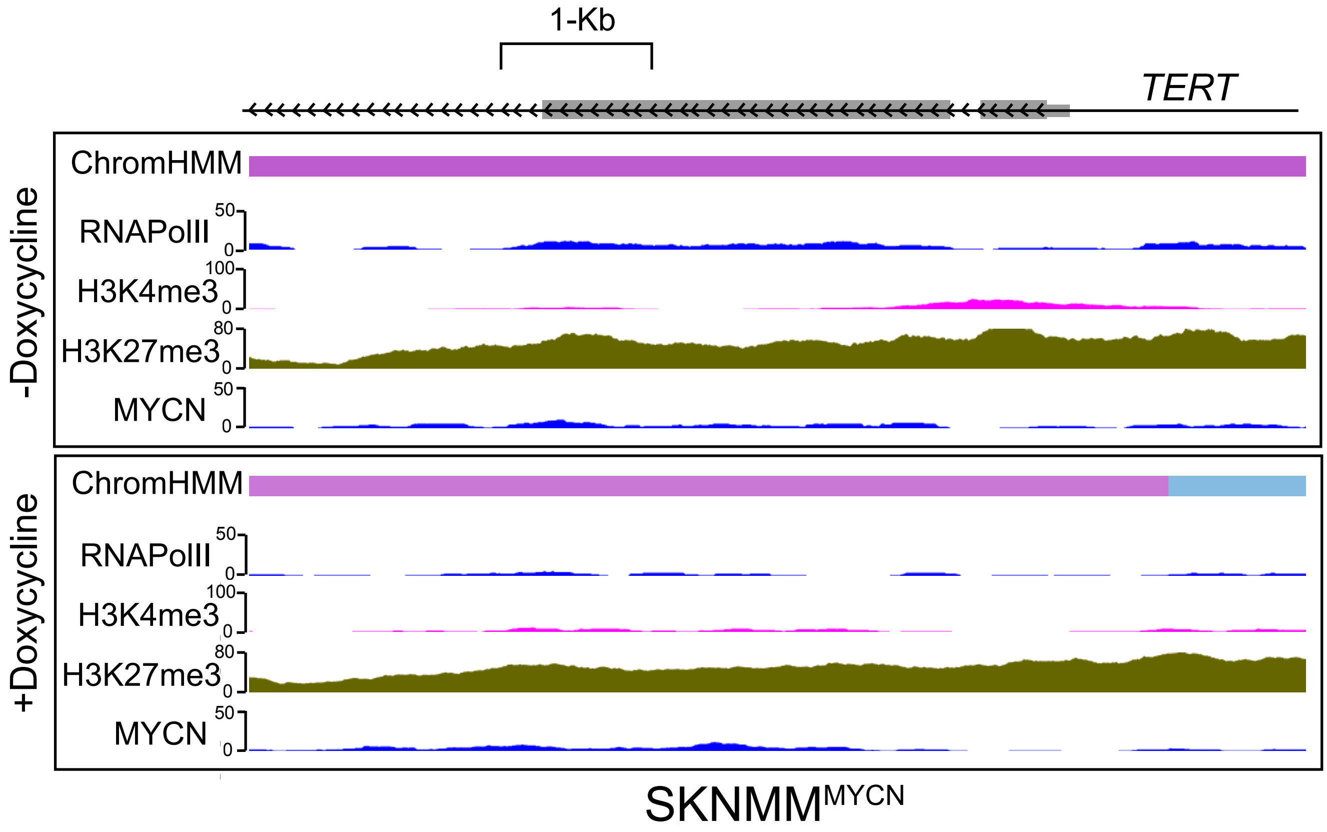
1-Kb
TERT
-Doxycycline
ChromHMM
RNAPolII
50
0
H3K4me3
100
0
H3K27me3
80
0
MYCN
50
0
+Doxycycline
ChromHMM
RNAPolII
50
0
H3K4me3
100
0
H3K27me3
80
0
MYCN
50
0
SKNMM MYCN

B

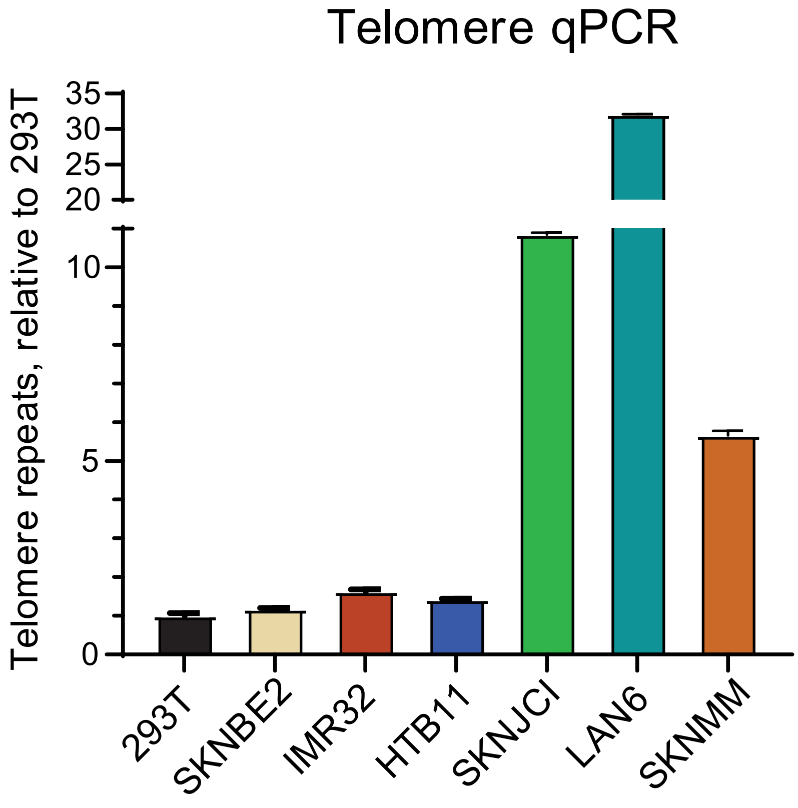
Telomere qPCR
Telomere repeats, relative to 293T
35
30
25
20
10
5
0
293T
SKNBE2
IMR32
HTB11
SKNJCI
LAN6
SKNMM

Supplementary figure S4: A) ChromHMM state and ChIP-Seq tracks for RNAPolII, H3K4me3, H3K27me3 and MYCN at the *TERT* locus of the doxycycline inducible MYCN-inducible neuroblastoma cells with long telomeres $SKNMM^{MYCN}$, with or without doxycycline. *TERT* locus is enriched for the repressive mark H3K27me3 and MYCN does not bind to the *TERT* promoter in these cells. **B)** Telomeric repeats in neuroblastoma cell line relative to those in the telomerase positive 293T cells using qPCR showing the neuroblastoma cells HTB11 have short telomeres while SKNJCI cells have long telomeres.
