# Supplementary material for: The TERT Promoter is Polycomb-Repressed in Neuroblastoma Cells with Long Telomeres
Source: Cancer Res Commun. 2024 Jun 20;4(6):1533–47. doi: 10.1158/2767-9764.CRC-22-0287 (PMC11188873; doi:10.1158/2767-9764.CRC-22-0287)
Supplement: Supplementary Figure S5 [file crc-22-0287-s05.pdf]

Figure S5

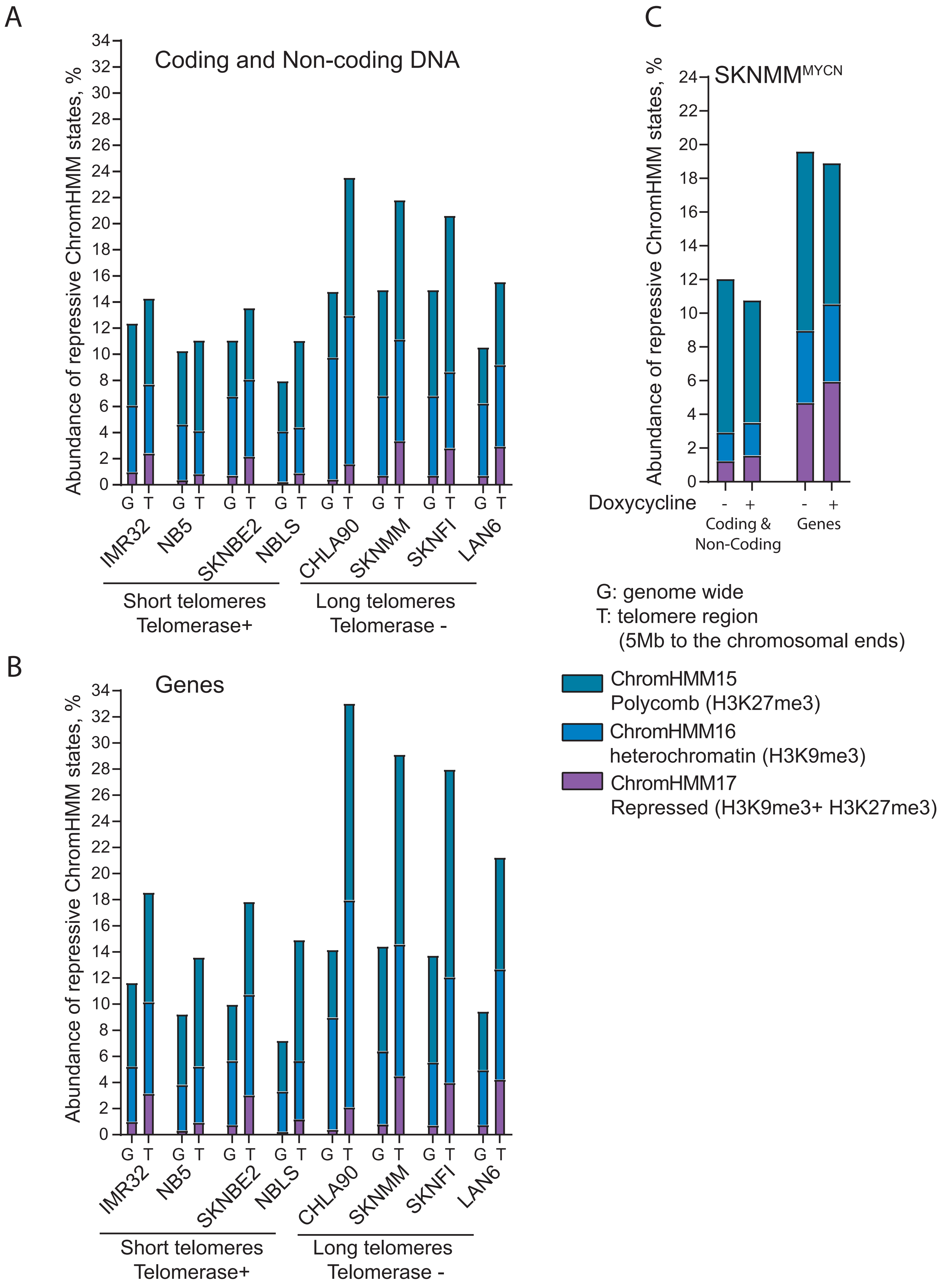

A
Coding and Non-coding DNA
Abundance of repressive ChromHMM states, %
34
32
30
28
26
24
22
20
18
16
14
12
10
8
6
4
2
0
G T G T G T G T G T G T G T G T
IMR32
NB5
SKNBE2
NBLS
CHLA90
SKNMM
SKNFI
LAN6
Short telomeres
Telomerase+
Long telomeres
Telomerase -
B
Genes
Abundance of repressive ChromHMM states, %
C
SKNMMMYCN
Abundance of repressive ChromHMM states, %
24
22
20
18
16
14
12
10
8
6
4
2
0
Doxycycline
- + - +
Coding & Non-Coding
Genes
G: genome wide
T: telomere region
(5Mb to the chromosomal ends)
ChromHMM15
Polycomb (H3K27me3)
ChromHMM16
heterochromatin (H3K9me3)
ChromHMM17
Repressed (H3K9me3+ H3K27me3)

Supplementary figure S5: Abundance of repressive chromatin ChromHMM states of 5 Mb subtelomeric region (T) and genome-wide (G) in neuroblastoma cells with short versus long telomeres. **A)** Abundance of repressive ChromHMM states in the whole coding and non-coding regions in different neuroblastoma cell lines. **B)** Abundance of repressive ChromHMM states at genic regions in different neuroblastoma cell lines. **C)** Repressive ChromHMM states at the chromosomal ends of a neuroblastoma cell line with long telomeres with or without induction of MYCN transgene.
