# Supplementary material for: The TERT Promoter is Polycomb-Repressed in Neuroblastoma Cells with Long Telomeres
Source: Cancer Res Commun. 2024 Jun 20;4(6):1533–47. doi: 10.1158/2767-9764.CRC-22-0287 (PMC11188873; doi:10.1158/2767-9764.CRC-22-0287)
Supplement: Supplementary Figure S6 [file crc-22-0287-s06.pdf]

Figure S6

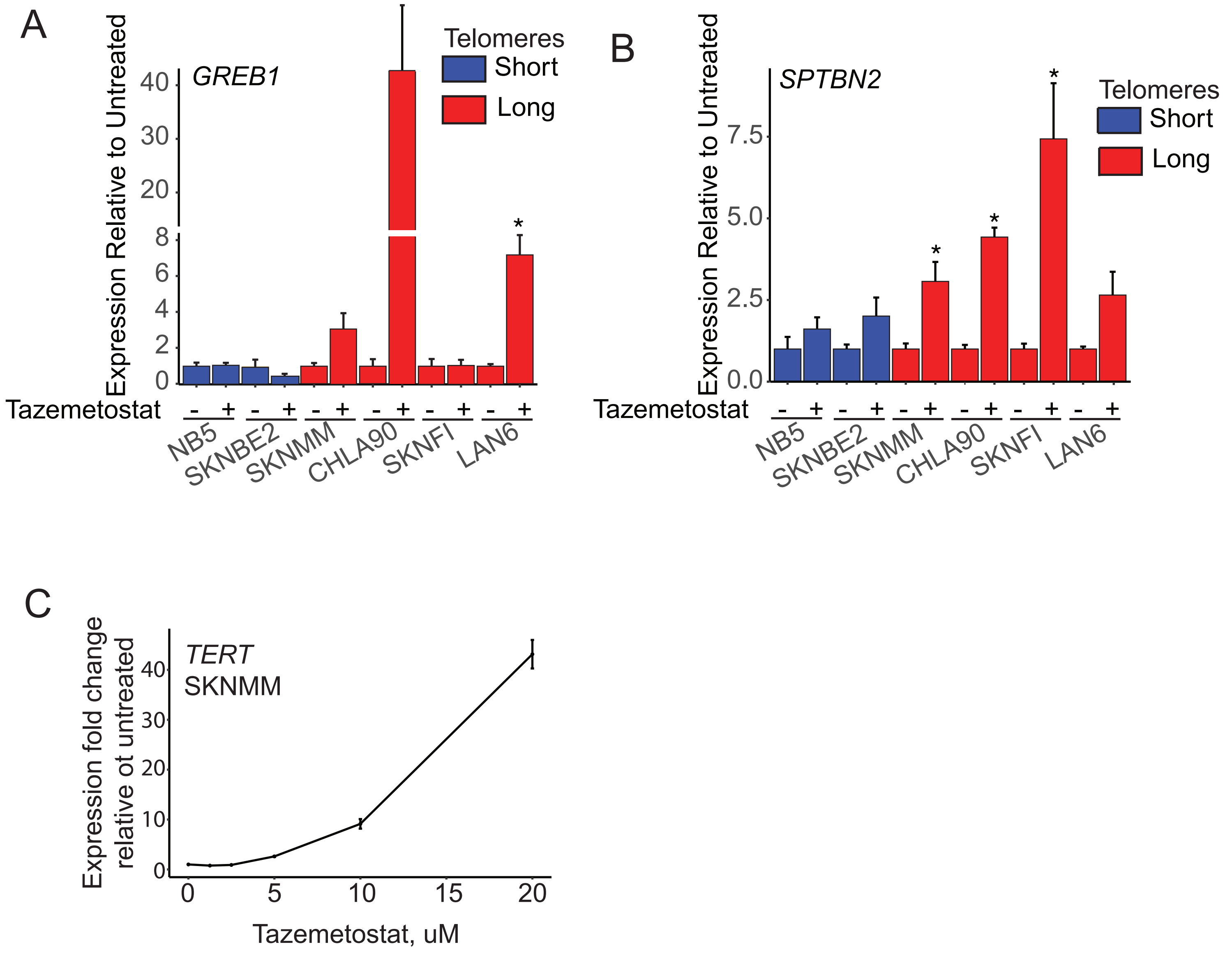

Supplementary figure S6: Fold change measured by RT-qPCR for **A)** *GREB1* and **B)** *SPTBN2* after treating neuroblastoma cell lines with tazemetostat or the drug vehicle only. **C)** Fold change measured by RT-qPCR for *TERT* in SKNMM cells treated with different concentration of the EZH2 inhibitor tazemetostat for 3 weeks.

* Indicates $p < 0.05$, error bars represent standard deviation.
