# Supplementary material for: The TERT Promoter is Polycomb-Repressed in Neuroblastoma Cells with Long Telomeres
Source: Cancer Res Commun. 2024 Jun 20;4(6):1533–47. doi: 10.1158/2767-9764.CRC-22-0287 (PMC11188873; doi:10.1158/2767-9764.CRC-22-0287)
Supplement: Supplementary Figure S7 [file crc-22-0287-s07.pdf]

Figure S7

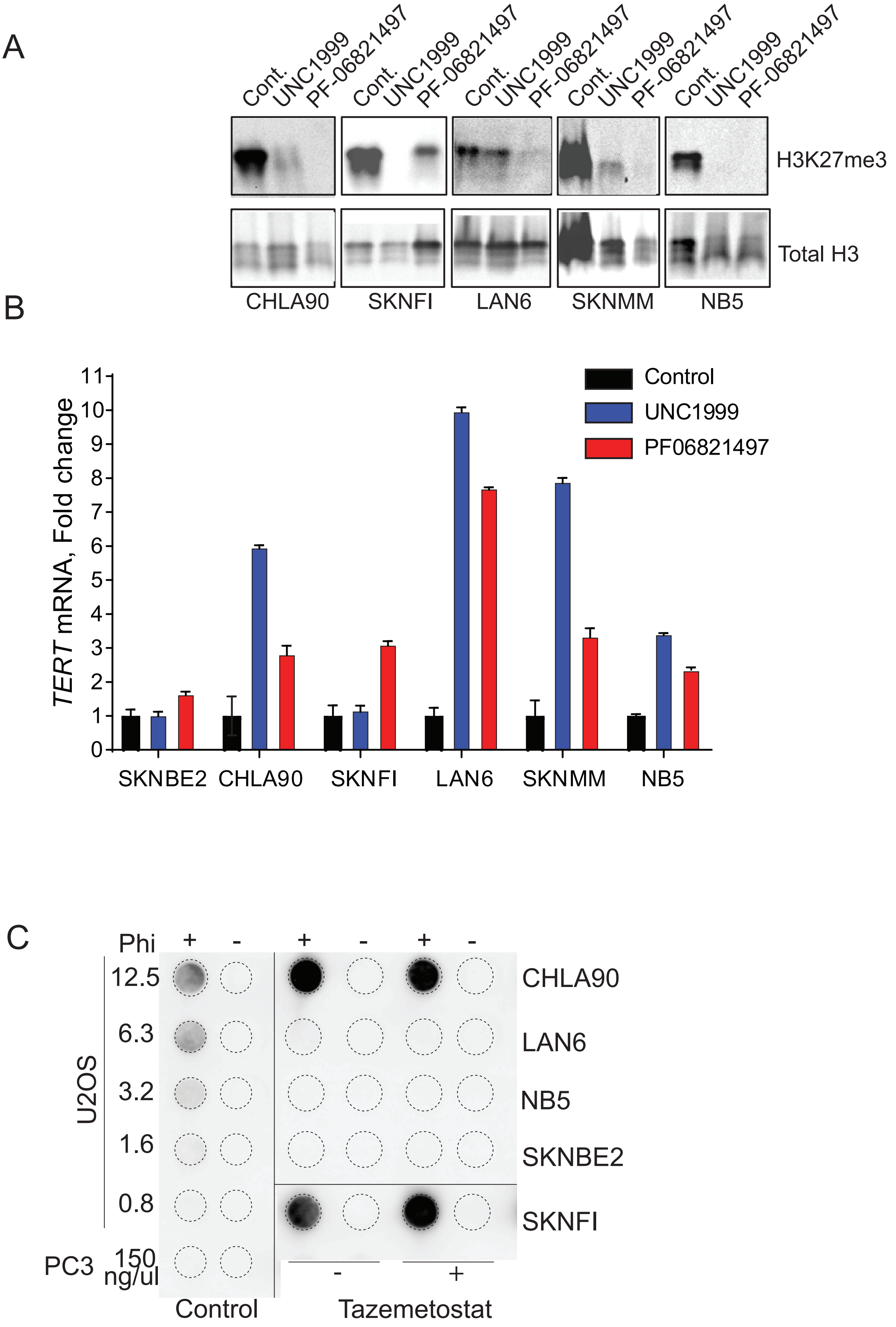

A
Cont.
UNC1999
PF-06821497
H3K27me3
Total H3
CHLA90
SKNFI
LAN6
SKNMM
NB5
B
TERT mRNA, Fold change
0
1
2
3
4
5
6
7
8
9
10
11
Control
UNC1999
PF06821497
SKNBE2
CHLA90
SKNFI
LAN6
SKNMM
NB5
C
Phi
+
-
U2OS
12.5
6.3
3.2
1.6
0.8
PC3
150 ng/ul
CHLA90
LAN6
NB5
SKNBE2
SKNFI
Control
Tazemetostat

Supplementary figure S7: A-B) Pharmacological inhibition of EZH2 using UNC1999 or PF-06821497 induces *TERT* expression in *TERT*-negative neuroblastoma cells. **A)** Western Blot for H3K27me3 and H3K27 total in neuroblastoma cells after treatment with UNC1999, PF-06821497 acetate or the drug vehicle only. **B**) Fold change of *TERT* expression in the same cells after the same treatment measured by qRT-PCR. **C)** C-circle assay blot with U2OS and PC3 cells used as positive and negative controls, respectively. The amount of DNA in nanograms (ng) is shown on the blot. All reactions were done in the presence or absence of the Phi 29 (Phi) DNA polymerase.
