# Supplementary material for: The TERT Promoter is Polycomb-Repressed in Neuroblastoma Cells with Long Telomeres
Source: Cancer Res Commun. 2024 Jun 20;4(6):1533–47. doi: 10.1158/2767-9764.CRC-22-0287 (PMC11188873; doi:10.1158/2767-9764.CRC-22-0287)
Supplement: Supplementary Figure S8 [file crc-22-0287-s08.pdf]

Figure S8

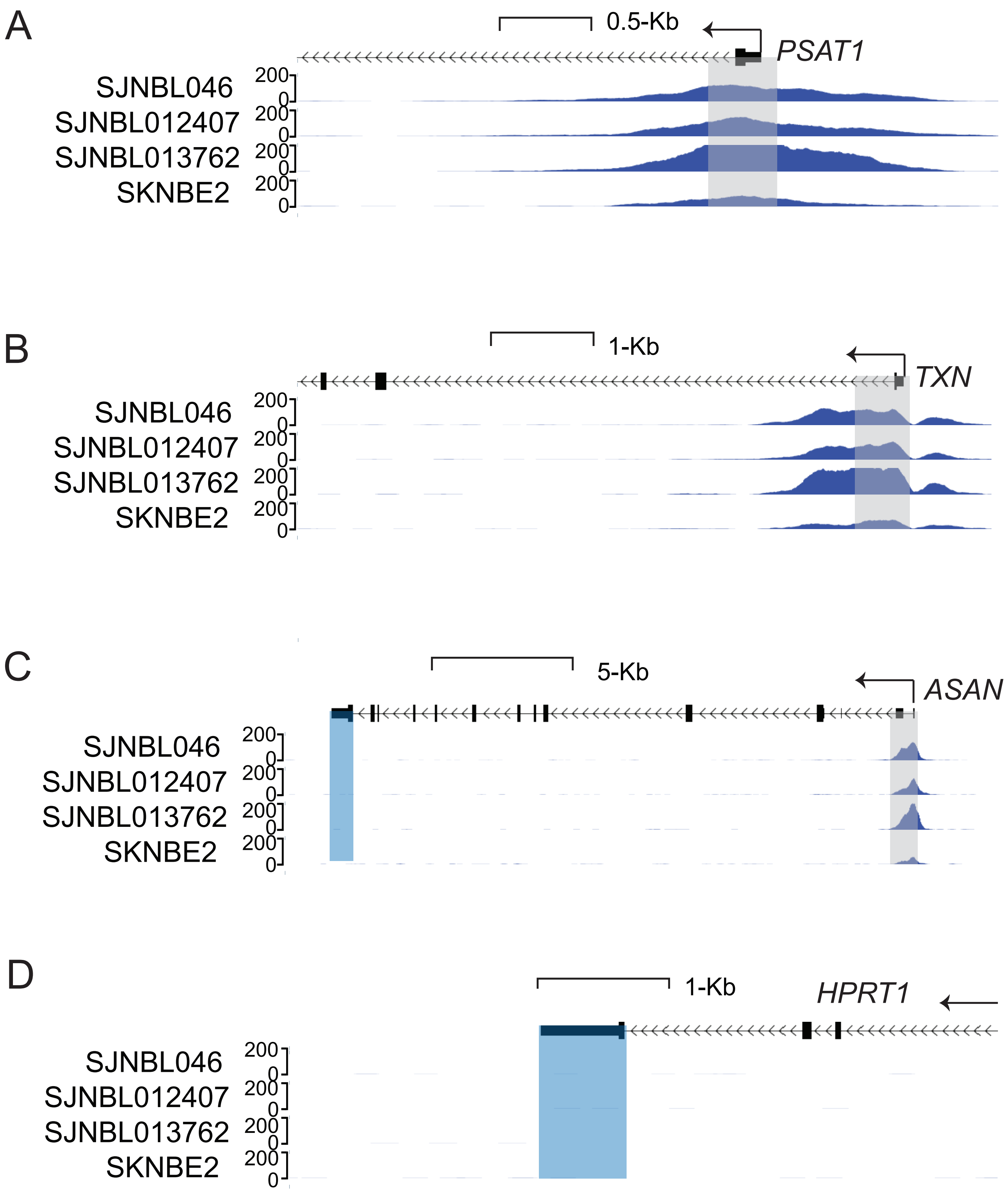
A
0.5-Kb
PSAT1
SJNBL046
SJNBL012407
SJNBL013762
SKNBE2
200
0
B
1-Kb
TXN
C
5-Kb
ASAN
D
1-Kb
HPRT1

Supplementary figure S8: Selecting positive and negative MYCN binding loci as controls for MYCN-ChIP qPCR assay. A-D) MYCN- ChIP-Seq in three MYCN-amplified neuroblastoma O-PDX models and cell line (SKNBE2) at the indicated genomic loci. Primers that target MYCN binding regions (highlighted in gray, **A-C**) and negative MYCN binding regions (highlighted in blue, **C-D**) were used for positive and negative controls respectively in MYCN ChIP qPCR assay.
