# Supplementary material for: The TERT Promoter is Polycomb-Repressed in Neuroblastoma Cells with Long Telomeres
Source: Cancer Res Commun. 2024 Jun 20;4(6):1533–47. doi: 10.1158/2767-9764.CRC-22-0287 (PMC11188873; doi:10.1158/2767-9764.CRC-22-0287)
Supplement: Supplementary tables — 1-4 [file crc-22-0287-s09.docx]

**Table S1.** Sequences of oligonucleotides used in this study.

| Oligonucleotide | Assay | Sequence (5’-3’) |
| --- | --- | --- |
| Telomere DIG Probe | C-circle Assay | CCCTAACCCTAACCCTAACCCTAA-DIG |
| HPRT_Forward | RT-qPCR, reference gene | CCTCCTCCTGAGCAGTCAGC |
| HPRT_Reverse | RT-qPCR, reference gene | CCCTTTCCAAATCCTCAGCA |
| TERT_Forward | RT-qPCR, target gene | AGCACCGTCTGCGTGAG |
| TERT_Reverse | RT-qPCR, target gene | CAGCTCGACGACGTACACAC |
| GREB1_Forward | RT-qPCR, target gene | ATGGGAAATTCTTACGCTGGAC |
| GREB1_Reverse | RT-qPCR, target gene | CACTCGGCTACCACCTTCT |
| SPTBN2_Forward | RT-qPCR, target gene | GAGGTCTCGCATTAAGGCTCT |
| SPTBN2_Reverse | RT-qPCR, target gene | CTTTGGCAGTATCTCTCCCGA |
| Telomere F | Telomere qPCR | CGGTTTGGGTTTGGGTTTGGGTTT GGGTTTGGGTTTGGGTTT |
| Telomere R | Telomere qPCR | GGCTTGCCTTACCCTTACCC  TTACCCTTACCCTTACCC |
| RPL0F | Telomere qPCR control locus | CAGCAAGTGGGAAGGTGTAATCC |
| RPL0R | Telomere qPCR control locus | CCCATTCTATCATCAACGGGTACAA |

**Supplementary table S2: primers used for MYCN-ChIP-qPCR**

| Primer name | Forward primer | Reverse primer | site |
| --- | --- | --- | --- |
| TERT1 | gaaaggaaggggaggggctg | cccttcaccttccagctccg | *TERT* promoter/ test |
| TERT2 | CCTCCCTGCAACACTTCCCC | TGGGGTGGTTTGCTCATGGT | *TERT* promoter/ test |
| ASNS1 | CCGACCTGGCTCCTGTAACG | CACTTAGTCCTGCTCCGCCC | *ASNS* promoter/ positive control |
| ASNS2 | AGATCAGGACCACGCGACAC | GCCTGTGGACATTTTCGCGG | *ASNS* promoter/ positive control |
| PSAT1 | CCTCCTTGGCTGACTCACCG | GCTCGCGGGGACTTACTGAG | *PSAT1*  promoter/ positive control |
| PSAT2 | AACGCGAGGAGGAGCAACTG | GCCCTGCGCTAATTGGTTCG | *PSAT1* promoter/ positive control |
| TXN | CTCACTTCCTCGCGGTCACC | AGGTGACGCGTGCTGTATCC | *TXN* promoter/ positive control |
| ASNS-Neg1 | ACCCACAGTCCCCATCCAAC | ATGCCACTGACCCTTCTGCC | *ASNS* last exon/ negative control |
| ASNS-Neg2 | CAGCTCTGCATCCAAACTGTCT | CCAAAAGAAGCCTTCAGTGATGG | *ASNS* last exon/ negative control |
| HPRT-Neg1 | GGAGGCAGAAGTCCCATGGA | GTGGGAGGCCAGTGTACGTG | *HPRT* last exon/ negative control |
| HPRT-Neg2 | TGTTTTCTCCTTCCAGCACCTCA | GTGTCACCCTAGCCTGGCC | *HPRT* last exon/ negative control |

­­

**Supplementary table S3A:** abundance of different ChromHMM in neuroblastoma cell lines.

**Genes** including 2Kb up and downstreams in the **5 Mb of subtelomeric** sequences.

|  | **Short telomeres** | | | | **Long telomeres** | | | |
| --- | --- | --- | --- | --- | --- | --- | --- | --- |
|  | **Telomerase +** | | | | **Telomerase -** | | | |
|  | **IMR32** | **NB5** | **SKNBE2** | **NBLS** | **CHLA90** | **SKNMM** | **SKNFI** | **LAN6** |
| **State1** | 1.31 | 0.54 | 1.24 | 1.46 | 1.18 | 1.42 | 1.22 | 1.42 |
| **State2** | 0.04 | 0.01 | 0.14 | 0.03 | 0.05 | 0.14 | 0.04 | 0.22 |
| **State3** | 1.13 | 0.88 | 2.31 | 1.06 | 0.66 | 0.77 | 0.63 | 2.10 |
| **State4** | 1.01 | 1.41 | 2.84 | 1.89 | 0.47 | 1.05 | 0.75 | 2.34 |
| **State5** | 1.94 | 3.36 | 1.40 | 2.32 | 2.13 | 1.81 | 1.26 | 1.36 |
| **State6** | 4.52 | 4.94 | 7.23 | 3.84 | 1.48 | 2.01 | 1.84 | 4.83 |
| **State7** | 0.45 | 0.65 | 1.04 | 0.25 | 0.56 | 1.05 | 0.49 | 0.60 |
| **State8** | 11.32 | 12.74 | 8.91 | 13.05 | 13.48 | 12.03 | 11.85 | 9.20 |
| **State9** | 7.43 | 5.95 | 3.65 | 6.06 | 2.83 | 8.27 | 8.56 | 6.16 |
| **State10** | 1.07 | 0.69 | 1.94 | 0.56 | 0.27 | 1.28 | 0.34 | 2.01 |
| **State11** | 2.08 | 1.31 | 1.93 | 1.09 | 0.47 | 0.84 | 0.96 | 1.41 |
| **State12** | 2.26 | 1.48 | 1.83 | 2.70 | 2.28 | 1.94 | 3.18 | 1.91 |
| **State13** | 0.09 | 0.08 | 0.18 | 0.10 | 0.04 | 0.28 | 0.04 | 0.26 |
| **State14** | 46.13 | 51.53 | 47.17 | 50.37 | 40.50 | 37.49 | 40.50 | 44.24 |
| **State15** | 8.38 | 8.36 | 7.11 | 9.26 | 15.06 | 14.54 | 15.90 | 8.55 |
| **State16** | 7.02 | 4.29 | 7.70 | 4.48 | 15.86 | 10.07 | 8.09 | 8.45 |
| **State17** | 3.16 | 0.94 | 3.04 | 1.19 | 2.11 | 4.51 | 3.99 | 4.24 |
| **State18** | 0.67 | 0.87 | 0.35 | 0.30 | 0.56 | 0.49 | 0.36 | 0.71 |

**Supplementary table S3B**: abundance of different ChromHMM in neuroblastoma cell lines.

**Genic and intergenic** regions at **5Mb of subtelomeric** sequences.

|  | **Short telomeres** | | | | **Long telomeres** | | | |
| --- | --- | --- | --- | --- | --- | --- | --- | --- |
|  | **Telomerase +** | | | | **Telomerase -** | | | |
|  | **IMR32** | **NB5** | **SKNBE2** | **NBLS** | **CHLA90** | **SKNMM** | **SKNFI** | **LAN6** |
| **State1** | 0.68 | 0.28 | 0.64 | 0.79 | 0.63 | 0.74 | 0.64 | 0.75 |
| **State2** | 0.02 | 0.00 | 0.07 | 0.01 | 0.03 | 0.08 | 0.02 | 0.12 |
| **State3** | 0.71 | 0.52 | 1.30 | 0.63 | 0.40 | 0.46 | 0.38 | 1.25 |
| **State4** | 0.69 | 0.86 | 1.72 | 1.20 | 0.28 | 0.64 | 0.47 | 1.51 |
| **State5** | 1.08 | 1.91 | 0.77 | 1.34 | 1.21 | 1.02 | 0.71 | 0.75 |
| **State6** | 2.87 | 3.07 | 4.54 | 2.49 | 0.89 | 1.28 | 1.15 | 3.17 |
| **State7** | 0.27 | 0.40 | 0.62 | 0.16 | 0.37 | 0.67 | 0.31 | 0.35 |
| **State8** | 5.83 | 6.58 | 4.60 | 6.94 | 7.11 | 6.17 | 6.11 | 4.81 |
| **State9** | 3.76 | 3.03 | 1.86 | 3.19 | 1.48 | 4.20 | 4.37 | 3.20 |
| **State10** | 0.54 | 0.35 | 0.98 | 0.29 | 0.14 | 0.66 | 0.18 | 1.05 |
| **State11** | 1.19 | 0.69 | 1.08 | 0.61 | 0.26 | 0.46 | 0.51 | 0.78 |
| **State12** | 1.23 | 0.80 | 0.99 | 1.50 | 1.27 | 1.07 | 1.71 | 1.06 |
| **State13** | 0.04 | 0.04 | 0.09 | 0.05 | 0.02 | 0.14 | 0.02 | 0.13 |
| **State14** | 66.35 | 69.78 | 66.95 | 69.53 | 62.00 | 60.27 | 62.57 | 65.03 |
| **State15** | 6.57 | 6.94 | 5.47 | 6.64 | 10.58 | 10.66 | 11.97 | 6.35 |
| **State16** | 5.29 | 3.29 | 5.90 | 3.49 | 11.36 | 7.77 | 5.82 | 6.24 |
| **State17** | 2.42 | 0.85 | 2.18 | 0.91 | 1.60 | 3.38 | 2.83 | 2.96 |
| **State18** | 0.45 | 0.60 | 0.24 | 0.22 | 0.37 | 0.33 | 0.24 | 0.49 |

**Supplementary table S3C**: abundance of different ChromHMM in neuroblastoma cell lines. **Genes** including 2Kb up and downstream, **genome-wide**.

|  | **Short telomeres** | | | | **Long telomeres** | | | |
| --- | --- | --- | --- | --- | --- | --- | --- | --- |
|  | **Telomerase +** | | | | **Telomerase -** | | | |
|  | **IMR32** | **NB5** | **SKNBE2** | **NBLS** | **CHLA90** | **SKNMM** | **SKNFI** | **LAN6** |
| **State1** | 0.77 | 0.37 | 0.69 | 0.83 | 0.84 | 0.90 | 0.71 | 0.84 |
| **State2** | 0.01 | 0.00 | 0.03 | 0.01 | 0.02 | 0.04 | 0.01 | 0.05 |
| **State3** | 0.58 | 0.68 | 0.87 | 0.44 | 0.64 | 0.59 | 0.60 | 0.89 |
| **State4** | 0.99 | 1.40 | 1.90 | 1.33 | 0.72 | 1.33 | 1.17 | 1.51 |
| **State5** | 1.27 | 2.23 | 1.05 | 1.62 | 1.61 | 1.68 | 1.05 | 1.18 |
| **State6** | 2.10 | 4.01 | 2.96 | 2.81 | 2.04 | 2.53 | 1.84 | 3.00 |
| **State7** | 0.19 | 0.33 | 0.37 | 0.12 | 0.25 | 0.42 | 0.25 | 0.21 |
| **State8** | 9.74 | 10.29 | 8.01 | 9.97 | 10.12 | 11.57 | 9.94 | 8.77 |
| **State9** | 4.51 | 3.03 | 2.01 | 2.37 | 0.96 | 3.57 | 4.99 | 3.77 |
| **State10** | 0.26 | 0.20 | 0.36 | 0.16 | 0.10 | 0.41 | 0.13 | 0.49 |
| **State11** | 0.70 | 0.61 | 0.61 | 0.40 | 0.14 | 0.27 | 0.35 | 0.44 |
| **State12** | 0.70 | 0.54 | 0.72 | 1.15 | 0.79 | 0.68 | 0.89 | 0.64 |
| **State13** | 0.04 | 0.03 | 0.05 | 0.02 | 0.02 | 0.09 | 0.02 | 0.07 |
| **State14** | 66.00 | 66.24 | 70.09 | 71.32 | 67.16 | 61.00 | 64.03 | 68.10 |
| **State15** | 6.42 | 5.40 | 4.31 | 3.88 | 5.18 | 8.02 | 8.18 | 4.48 |
| **State16** | 4.22 | 3.49 | 4.91 | 3.09 | 8.58 | 5.60 | 4.82 | 4.20 |
| **State17** | 1.01 | 0.33 | 0.76 | 0.24 | 0.40 | 0.81 | 0.73 | 0.76 |
| **State18** | 0.50 | 0.81 | 0.27 | 0.24 | 0.44 | 0.49 | 0.28 | 0.60 |

**Supplementary table S3D**: abundance of different ChromHMM in neuroblastoma cell lines. **Genic and intergenic** regions, **genome-wide**.

|  | **Short telomeres** | | | | **Long telomeres** | | | |
| --- | --- | --- | --- | --- | --- | --- | --- | --- |
|  | **Telomerase +** | | | | **Telomerase -** | | | |
|  | **IMR32** | **NB5** | **SKNBE2** | **NBLS** | **CHLA90** | **SKNMM** | **SKNFI** | **LAN6** |
| **State1** | 0.56 | 0.27 | 0.51 | 0.63 | 0.62 | 0.64 | 0.52 | 0.61 |
| **State2** | 0.01 | 0.00 | 0.02 | 0.01 | 0.01 | 0.03 | 0.01 | 0.03 |
| **State3** | 0.51 | 0.58 | 0.74 | 0.40 | 0.57 | 0.51 | 0.54 | 0.78 |
| **State4** | 0.96 | 1.28 | 1.78 | 1.28 | 0.66 | 1.21 | 1.13 | 1.42 |
| **State5** | 1.00 | 1.79 | 0.83 | 1.34 | 1.32 | 1.33 | 0.87 | 0.91 |
| **State6** | 1.93 | 3.65 | 2.70 | 2.73 | 1.83 | 2.25 | 1.74 | 2.78 |
| **State7** | 0.15 | 0.26 | 0.30 | 0.10 | 0.21 | 0.35 | 0.21 | 0.17 |
| **State8** | 6.97 | 7.26 | 5.88 | 7.41 | 7.37 | 8.08 | 7.14 | 6.32 |
| **State9** | 3.19 | 2.11 | 1.46 | 1.73 | 0.69 | 2.46 | 3.53 | 2.68 |
| **State10** | 0.18 | 0.14 | 0.27 | 0.12 | 0.07 | 0.29 | 0.10 | 0.35 |
| **State11** | 0.57 | 0.46 | 0.50 | 0.33 | 0.11 | 0.21 | 0.27 | 0.34 |
| **State12** | 0.54 | 0.40 | 0.56 | 0.90 | 0.60 | 0.51 | 0.67 | 0.49 |
| **State13** | 0.03 | 0.02 | 0.04 | 0.02 | 0.01 | 0.06 | 0.01 | 0.05 |
| **State14** | 70.52 | 70.69 | 73.04 | 74.79 | 70.69 | 65.90 | 68.02 | 71.91 |
| **State15** | 6.28 | 5.64 | 4.30 | 3.85 | 5.04 | 7.77 | 8.13 | 4.30 |
| **State16** | 5.10 | 4.26 | 6.05 | 3.87 | 9.34 | 7.08 | 6.09 | 5.54 |
| **State17** | 0.99 | 0.38 | 0.73 | 0.24 | 0.43 | 0.83 | 0.73 | 0.71 |
| **State18** | 0.51 | 0.82 | 0.28 | 0.26 | 0.44 | 0.49 | 0.28 | 0.61 |

**Supplemental table S4A: ChIP-qPCR enrichment for MYCN**

| **Primers** | **SKNBE** | | **NB5** | | | | **HTB11- MYCN** | | **HTB11- MYCN-DOX** | | **SKNMM- MYCN** | | |
| --- | --- | --- | --- | --- | --- | --- | --- | --- | --- | --- | --- | --- | --- |
|  | **Enrich, %** | **SD** | | **Enrich, %** | **SD** | **Enrich, %** | | **SD** | **Enrich, %** | **SD** | | **Enrich, %** | **SD** |
| **TERT1** | 0.3032 | 0.0594 | | 0.1576 | 0.0255 | 0.0663 | | 0.0074 | 0.2097 | 0.0001 | | 0.0619 | 0.0043 |
| **TERT2** | 0.2372 | 0.0406 | | 0.1188 | 0.0018 | 0.0080 | | 0.0006 | 0.1925 | 0.0027 | | 0.0083 | 0.0006 |
| **ASNS1** | 0.2601 | 0.0124 | | 0.2202 | 0.0195 | 0.0323 | | 0.0017 | 0.0641 | 0.0048 | | 0.0101 | 0.0013 |
| **ASNS2** | 0.3090 | 0.0387 | | 0.3455 | 0.0592 | 0.0230 | | 0.0006 | 0.0671 | 0.0012 | | 0.0649 | 0.0009 |
| **PSAT1** | NA | NA | | 0.5138 | 0.0393 | 0.0087 | | 0.0001 | 0.2572 | 0.0136 | | 0.2355 | 0.0193 |
| **PSAT2** | 0.4760 | 0.0430 | | 0.5437 | 0.0485 | 0.0112 | | 0.0012 | 0.2880 | 0.0074 | | 0.1126 | 0.0053 |
| **TXN** | 0.0889 | 0.0072 | | 0.0625 | 0.0110 | 0.0071 | | 0.0003 | 0.0027 | 0.0005 | | 0.0006 | 0.0001 |
| **ASNS-Neg1** | 0.0048 | 0.0001 | | 0.0062 | 0.0008 | 0.0106 | | 0.0002 | 0.0056 | 0.0005 | | 0.0065 | 0.0006 |
| **ASNS-Neg2** | 0.0020 | 0.0002 | | 0.0042 | 0.0001 | 0.0056 | | 0.0010 | 0.0052 | 0.0002 | | 0.0081 | 0.0032 |
| **HPRT-Neg1** | 0.0020 | 0.0004 | | 0.0061 | 0.0003 | 0.0059 | | 0.0010 | 0.0062 | 0.0015 | | 0.0080 | 0.0009 |
| **HPRT-Neg2** | 0.0028 | 0.0010 | | 0.0047 | 0.0008 | 0.0059 | | 0.0010 | 0.0062 | 0.0008 | | 0.0086 | 0.0011 |

**Supplemental table S4B: ChIP-qPCR enrichment for MYCN**

| **Primers** | **SKNMM- MYCN-DOX** | | **SKNMM-MYCN-TMZ- DOX** | | **SKNJCI-MYCN** | | **JCI-MYCN-DOX** | | **JCI-MYCN-TMZ-DOX** | |
| --- | --- | --- | --- | --- | --- | --- | --- | --- | --- | --- |
|  | **Enrich, %** | **SD** | **Enrich, %** | **SD** | **Enrich, %** | **SD** | **Enrich, %** | **SD** | **Enrich, %** | **SD** |
| **TERT1** | 0.0576 | 0.0011 | 0.1261 | 0.0085 | 0.0860 | 0.0447 | 0.0959 | 0.0274 | 0.1974 | 0.0242 |
| **TERT2** | 0.0471 | 0.0004 | 0.1794 | 0.0196 | 0.0068 | 0.0015 | 0.0604 | 0.0032 | 0.1255 | 0.0127 |
| **ASNS1** | 0.1404 | 0.0017 | 0.1480 | 0.0022 | 0.0072 | 0.0015 | 0.1418 | 0.0043 | 0.1109 | 0.0019 |
| **ASNS2** | 0.1591 | 0.0124 | 0.1765 | 0.0200 | 0.0747 | 0.0041 | 0.2274 | 0.0099 | 0.1992 | 0.0013 |
| **PSAT1** | 0.2951 | 0.0062 | 0.3627 | 0.0121 | 0.1418 | 0.0003 | 0.4549 | 0.0380 | 0.4334 | 0.0043 |
| **PSAT2** | 0.1933 | 0.0064 | 0.3359 | 0.0100 | 0.0389 | 0.0016 | 0.6243 | 0.0466 | 0.3340 | 0.0329 |
| **TXN** | 0.1300 | 0.0015 | 0.2154 | 0.0030 | 0.0328 | 0.0049 | 0.2636 | 0.0044 | 0.2648 | 0.0083 |
| **ASNS-Neg1** | 0.1102 | 0.0017 | 0.0045 | 0.0006 | 0.0032 | 0.0011 | 0.0063 | 0.0013 | 0.0058 | 0.0004 |
| **ASNS-Neg2** | 0.0091 | 0.0004 | 0.0108 | 0.0001 | 0.0070 | 0.0049 | 0.0068 | 0.0023 | 0.0064 | 0.0004 |
| **HPRT-Neg1** | 0.0218 | 0.0017 | 0.0091 | 0.0002 | 0.0070 | 0.0030 | 0.0068 | 0.0021 | 0.0064 | 0.0012 |
| **HPRT-Neg2** | 0.0162 | 0.0016 | 0.0063 | 0.0011 | 0.0042 | 0.0026 | 0.0075 | 0.0003 | 0.0091 | 0.0000 |

**Supplemental table S4C: ChIP-qPCR enrichment for MYCN**

| **Primers** | **U251-MYCN** | | **U251-MYCN-DOX** | | | **MOG-MYCN** | | **MOG-MYCN-DOX** | | **IgG** | |
| --- | --- | --- | --- | --- | --- | --- | --- | --- | --- | --- | --- |
|  | **Enrich, %** | **SD** | | **Enrich, %** | **SD** | **Enrich, %** | **SD** | **Enrich, %** | **SD** | **Enrich, %** | **SD** |
| **TERT1** | 0.0749 | 0.0254 | | 0.7333 | 0.1392 | 0.0353 | 0.0019 | 1.0394 | 0.1986 | 0.0475 | 0.0207 |
| **TERT2** | 0.0081 | 0.0002 | | 0.2676 | 0.0078 | 0.1056 | 0.0009 | 1.1023 | 0.1606 | 0.0015 | 0.0197 |
| **ASNS1** | 0.0323 | 0.0016 | | 0.3496 | 0.0030 | 0.0349 | 0.0009 | 1.2114 | 0.1482 | 0.0007 | 0.0138 |
| **ASNS2** | 0.0542 | 0.0014 | | 0.1744 | 0.0062 | 0.0263 | 0.0003 | 1.1127 | 0.1415 | 0.0013 | 0.0056 |
| **PSAT1** | 0.0581 | 0.0012 | | 0.9818 | 0.0041 | 0.0445 | 0.0058 | 0.8982 | 0.1170 | 0.0013 | 0.0337 |
| **PSAT2** | 0.0051 | 0.0006 | | 0.8556 | 0.0206 | 0.0048 | 0.0002 | 0.8566 | 0.1407 | 0.0012 | 0.0526 |
| **TXN** | 0.0057 | 0.0005 | | 0.0942 | 0.0030 | 0.0043 | 0.0006 | 0.3149 | 0.0437 | 0.0013 | 0.0106 |
| **ASNS-Neg1** | 0.0085 | 0.0003 | | 0.0067 | 0.0008 | 0.0047 | 0.0002 | 0.0122 | 0.0023 | 0.0020 | 0.0069 |
| **ASNS-Neg2** | 0.0096 | 0.0005 | | 0.0165 | 0.0003 | 0.0091 | 0.0010 | 0.0174 | 0.0007 | 0.0010 | 0.0191 |
| **HPRT-Neg1** | 0.0096 | 0.0005 | | 0.0165 | 0.0026 | 0.0091 | 0.0009 | 0.0174 | 0.0010 | 0.0010 | 0.0093 |
| **HPRT-Neg2** | 0.0061 | 0.0025 | | 0.3217 | 0.0074 | 0.0030 | 0.0010 | 0.0492 | 0.0052 | 0.0013 | 0.0487 |

**Supplemental table S4D: ChIP-qPCR enrichment for CTCF**

| **Primers** | CTCF | | IgG | |
| --- | --- | --- | --- | --- |
|  | **Enrich, %** | **SD** | **Enrich, %** | **SD** |
| **Myoglobin** | 0.0933 | 0.0087 | 0.0013 | 0.0001 |
| **H19** | 0.8474 | 0.0942 | 0.0016 | 0.0009 |

­­
